# Unary Adsorption Equilibria of Hydrogen, Nitrogen, and Carbon Dioxide on Y-Type Zeolites at Temperatures from 298 to 393 K and at Pressures up to 3 MPa

**DOI:** 10.1021/acs.jced.3c00504

**Published:** 2023-10-30

**Authors:** Hassan Azzan, David Danaci, Camille Petit, Ronny Pini

**Affiliations:** Department of Chemical Engineering, Imperial College London, London SW7 2AZ, United Kingdom

## Abstract

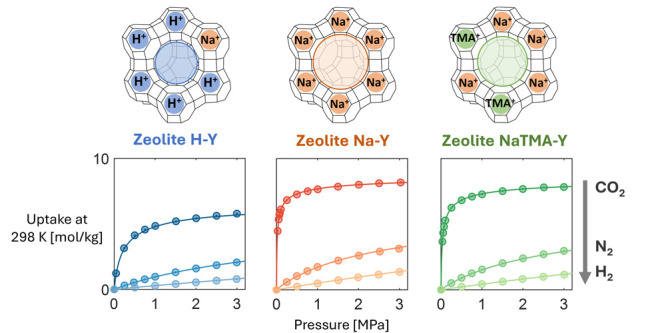

The equilibrium adsorption of CO_2_, N_2_, and H_2_ on commercially available Zeolite H–Y, Na–Y, and cation-exchanged NaTMA–Y was measured up to 3 MPa at 298.15, 313.15, 333.15, 353.15, and 393.15 K gravimetrically using a magnetic suspension balance. The chemical and textural characterization of the materials was carried out by thermogravimetric analysis, helium gravimetry, and N_2_ (77 K) physisorption. We report the excess and net isotherms as measured and estimates of the absolute adsorption isotherms. The latter are modeled using the simplified statistical isotherm (SSI) model to evaluate adsorbate–adsorbent interactions and parametrize the data for process modeling. When reported per unit volume of zeolite supercage, the SSI model indicates that the saturation capacity for a given gas takes the same value for the three adsorbents. The Henry’s constants predicted by the model show a strong effect of the cation on the affinity of each adsorbate.

## 1 Introduction

Carbon dioxide (CO_2_) emissions from fossil fuel combustion and industrial processes accounted for 64% of all net anthropogenic greenhouse gas emissions in 2019.^1^ Over the past few years, unit costs of both low emission and renewable energy technologies, such as photovoltaic cells and batteries, have declined and have led to increased adoption.^2^ However, these technologies do not directly aid in abating CO_2_ emissions from large point sources, and additional technological solutions need to be developed and implemented into new and existing infrastructure to reduce these emissions and to limit global warming to below 2 °C by 2050.

By targeting CO_2_ emissions in a variety of sectors, including both primary (*e.g*., power generation, steel making, cement industry) and secondary emitters (*e.g*., waste incinerators and chemical plants), postcombustion carbon capture constitutes a key technology that can contribute to this objective.^3^ Here, the key separation is between CO_2_ and nitrogen (N_2_), which are the primary components of the dry flue gas. Such streams are typically at or near ambient temperatures and pressures, and can compose of a wide range of CO_2_ content depending on the upstream process (5–15%_vol_ CO_2_ concentration).^4^ Another avenue for industrial decarbonization is precombustion capture, a separation technology integrated within the production of low-carbon hydrogen (H_2_) to be used as a feed-stock for chemical processes, *e.g*., ammonia production, or as an alternative fuel to natural gas. In this case, the key separation is between the CO_2_ and H_2_. The current state-of-the-art for H_2_ production is via coal or natural gas reforming reactions, yielding a gas mixture rich in H_2_ and with a concentration of CO_2_ that can range from 15–50%_vol_ (the remaining components being methane, CH_4_, and carbon monoxide, CO).^5^

Adsorption-based processes are being extensively researched for the separations mentioned above. Assessing and optimizing the feasibility of adsorption-based carbon capture technologies require computational tools, such as numerical process models of varying complexity.^6−8^ Irrespective of the approach used, the necessary input to these models is empirical or predictive knowledge of the adsorption equilibria of the relevant gases under relevant process conditions (pressure and temperature) for a given adsorbent material. Where data are not readily available, as it is the case for multicomponent adsorption, predictive models are used to describe the competitive behavior between species in a gas mixture.^9−12^ In this case, formulations that are commonly applied rely on accurate descriptions of unary adsorption isotherms.

The thermodynamics of adsorption depend fundamentally on the interaction between the surface of the solid sorbent and the bulk adsorbate. Adsorption-based separation processes exploit the differences in these interactions between two or more species in the bulk to achieve a separation.^13^ Zeolites are one of the most versatile classes of adsorbent materials which are also used widely in catalysis and chemical separations.^14^ Faujasite (FAU) zeolites in particular, *e.g*., Zeolite Y, carry promise in their application to separations, owing to their highly stable crystalline structure which can withstand extreme temperatures, as well as their large micropore volumes which enhance the adsorption capacity.^15^ FAU zeolites also possess strong cation exchange capacity due to the low silicon/aluminum atomic ratio (Si/Al), which can be exploited to tune their adsorption properties for different applications.^15^ Shiralkar and Kulkarni^16^ assessed the effect of intrazeolitic cations (La^3+^, Ca^2+^, and H^+^) on CO_2_ adsorption in Y-type zeolites and proposed differences in the state of the adsorbed molecules using different isotherm model formulations. Walton et al.^17^ described the effect of alkali metal cation exchange on CO_2_ adsorption on zeolite Na–X (Si/Al = 1.23) and Na–Y (Si/Al = 2.35) and found wide variations in the total CO_2_ adsorption capacity and Henry’s law constants upon cation exchange at 298 K and below 100 kPa, in addition to a greater extent of exchange for Na–Y compared to Na–X for all alkali metal cations. For a commercial Na–Y zeolite, Feng et al.^18^ report the equilibrium adsorption capacity to follow the order CO_2_ ≫ CH_4_ > CO > N_2_ ≫ H_2_ for measurements up to 1 MPa in the temperature range 298 to 358 K. The use of the organic cation tetramethylammonium (TMA) exchanged Na–Y for the separation of CH_4_/N_2_ has also been evaluated in different studies by Li et al.,^19^ Avijegon,^20^ Wu et al.,^21^ and Sadeghi Pouya et al.,^22^ showing an improvement in selectivity of CH_4_ over N_2_ when compared with Na–Y. However, this form of zeolite Y has not been studied for pre- and postcombustion carbon capture applications.

In this work, we present unary gas adsorption isotherm data on Y-type zeolites (Si/Al = 2.55), namely, on the commercially available Zeolite H–Y and Na–Y along with NaTMA–Y produced by cation exchange on the commercial Na–Y. We carried out these measurements gravimetrically over a wide range of temperatures and pressures relevant to pre- and postcombustion CO_2_ capture. Specifically, we present the adsorption of CO_2_, N_2_, and H_2_ up to 3 MPa in the temperature range from 298.15 to 393.15 K. We describe the obtained isotherm data with the simplified statistical isotherm model first developed by Ruthven^23^ and extract useful properties that can help elucidate the impact of the cation on the adsorption properties. In presenting the adsorption data, we demonstrate important protocols required for the measurement and modeling of adsorption equilibrium data on zeolites and highlight sources of uncertainty in the experimental data and model parameters.

## 2 Materials and Methods

### 2.1 Materials and Gases

The full list of materials, reagents, and gases used in this work is provided in Table 1. The adsorbent materials Zeolite H–Y and Na–Y were used for all the measurements in their crystalline form as supplied. These adsorbents are Y zeolites with FAU unit cells and exhibit the same Si:Al ratio (Si:Al = 2.55). The gases used in this study (He, CO_2_, and N_2_) were used as supplied without additional purification. H_2_ was produced using a PEAK Scientific Precision Hydrogen 100 H_2_ generator at a maximum pressure of 0.8 MPa and boosted up to 3 MPa using a Maximator DLE 5-1-2 GG hydrogen gas booster as described in our previous work.^24^

**Table 1:** Details of the Adsorbent Materials, Reagents, and Gases Used in This Study, as Provided by the Manufacturer/Supplier

name	CAS no.	source	purity [%]
Zeolite H–Y powder (CBV400)	1318-02-1	Zeolyst	100
Zeolite Na–Y powder (RM8850)	1318-02-1	NIST	100
tetramethylammonium chloride (reagent grade)	75-57-0	Sigma-Aldrich	≥98
carbon dioxide (CO_2_)	124-38-9	BOC	99.995
nitrogen (N_2_)	7727-37-9	BOC	99.9992
hydrogen (H_2_)	1333-74-0	PEAK Scientific	99.9995
helium (He)	7440-59-7	BOC	99.999

Zeolite NaTMA–Y was obtained by conducting cation exchange on Zeolite Na–Y using the following procedure. Two grams of Zeolite Na–Y was mixed with 100 mL of 1 M tetramethylammonium chloride in a glass bottle along with a magnetic stirrer, placed in an oil bath atop a stirrer plate, and heated to 353 K for 12 h. The solid was then allowed to settle, and the solution was decanted and replenished with 100 mL of 1 M tetramethylammonium chloride solution. The 12 h reaction was repeated a total of four times. Following this, the mixture was transferred to centrifuge tubes, and the solid was separated by centrifugation at 3000 rpm for 3 min. The liquid was decanted, and the tubes were topped up to 40 mL with DI water and agitated followed again by centrifugation. This washing step is critical in removing any residual salt and was repeated five times. The resulting slurry after decantation was dried in a vacuum oven at 473 K for 16 h. Zeolite NaTMA–Y was activated at 623 K under vacuum for 16 h.

### 2.2 Determination of Chemical and Textural Parameters

The TMA content in Zeolite NaTMA–Y prior to and after activation was determined by thermogravimetric analysis (TGA) using a commercial instrument (Netzsch TG 209 F1 Libra) under constant air flow at 80 mL min^–1^, with N_2_ as balance protection gas at 20 mL min^–1^. The temperature was ramped from ambient temperature to 1150 at 10 K min^–1^. The mass loss above 625 K, *i.e*., the temperature at which all the residual moisture was removed, was attributed to thermal decomposition of TMA^+^.

Textural analysis on the three adsorbent samples was carried out by N_2_ physisorption at 77 K performed using a commercial Autosorb iQ (Quantachrome Instruments) instrument in the relative pressure range 5 × 10^–7^ to 0.99. The samples were activated *ex-situ* prior to the volumetric measurements using the following protocol: (1) 1 h at 323 K; (2) 2 h at 373 K; and (3) 16 h at 623 K. The pore volume and surface area of the samples were determined by fitting the equilibrium isotherms with the nonlocal density functional theory (NLDFT) model for zeolite/silica cylindrical/spherical pores using the proprietary software (ASiQwin), with the resulting fitting error being <2%. BET area was calculated using the multipoint method following the Rouquerol criteria^25^ using the open-source software package BETSI (BET Surface Identification) constraining the coefficient of determination (*R*^2^) to a minimum of 0.995.^26^

### 2.3 Equilibrium Adsorption Measurements

High pressure gravimetric adsorption measurements were conducted by using a commercial two-position Rubotherm magnetic suspension balance (MSB) (Isosorp HPII). The details of the setup, and operating procedure have been described in our previous works.^24,27^ In this work, the samples were heated *in situ* to 623 K under a minimum pressure of 4 × 10^–4^ mbar at a ramp rate of 1 K min^–1^ and activated for at least 16 h prior to any measurement.

The net and excess amount adsorbed can be measured using this setup by using the first measurement point, MP_1_ [g], to weigh the sample plus the suspended metal parts, and the second measurement point, MP_2_ [g], to weigh MP_1_ plus the weight of a titanium sinker of a known volume, *V*_sk_ = 4.364 ± 0.002 cm^3^. For a sample of fixed volume *V*_s_ [cm^3^], at a given bulk density of fluid, ρ_b_ [g cm^–3^], and temperature, *T* [K], the gravimetric net and excess amount adsorbed are defined as
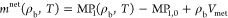
1
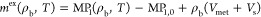
2where *V*_met_ = 1.420 ± 0.001 cm^3^ is the calibrated volume of the suspended metal components. The bulk density of the fluid is measured *in situ* and calculated using
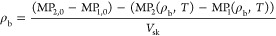
3where the subscript “0” refers to the weight measurements under vacuum. For the case of H_2_, gas density values were obtained at relevant *P* and *T* conditions via the NIST Chemistry Web Book^28^ which tabulates thermophysical properties for H_2_ calculated using the equation of state developed by Leachman et al.^29^ The reason for not using the *in situ* measured bulk density values is attributed to the low density of the H_2_ at the experimental conditions. The latter resulted in the sinker’s weight change due to buoyancy effects being consistently within 10 times the resolution of the instrument, yielding large fluctuations over different measurements. The *in situ* measured values for ρ_b_ as a function of pressure compared with the equation of state values for CO_2_ and N_2_ for the three materials are shown in Figure S1 in the Supporting Information.

The value of *V*_met_ was obtained by calibration of the system via measurements using CO_2_ in the absence of any adsorbing material at 353 K starting in a vacuum state and increasing in pressure up to the maximum pressure probed in the experiments. The sample volume plus the volume of the metal parts (*V*_0_ = *V*_met_ + *V*_s_) required for the calculation of *m*^ex^ was measured by helium gravimetry following the same procedure by starting from a vacuum state at a temperature of 393 K and loading the sample cell with helium to a maximum of 3 MPa. This was described in detail by Pini et al.^30^ From this, the skeletal density of the solid can be calculated using the following equation:
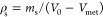
4where *m*_s_ [g] is the sample mass. Following a strict activation protocol is important for an accurate measurement of the skeletal density and resulting excess isotherms, particularly for low Si/Al zeolites such as those used in this study due to their hygroscopic nature.^31−33^ Ensuring the purity and minimizing the moisture content in the gases used is also paramount for these materials, and the validity of using a H_2_ generator for these measurements was discussed and established in our previous work.^24^

The equilibrium measurements were carried out starting from a vacuum state (a minimum pressure of 4 × 10^–8^ MPa) at a constant temperature in order to obtain reference values for the two measurement points, MP_1,0_ and MP_2,0_, respectively. Following this, the pressure was increased to the next point on the isotherm, and the sample was allowed to equilibrate at the fixed temperature. The system was left to reach equilibrium for at least 60 min, until the standard deviation in the corrected MP_1_ was below 50 μg for at least 25 min. Overnight (≥12 h) measurements were regularly conducted to confirm that no significant amounts of adsorption occur past the usual equilibration time. After reaching equilibrium, the average of the last five measurements was used to calculate the adsorption amount at the given pressure and temperature conditions. Tests were carried out to ensure no hysteresis in the measurements (at least one equilibrium data point was measured in both adsorption and desorption modes for CO_2_ at an intermediate pressure with 60 min equilibration). The standard method was to measure the isotherms using a desorption protocol (pressure raised to the maximum measurement pressure after completion of the vacuum point and then reduced for every point on the isotherm) to reduce temperature equilibration times. The measurement accuracy of the experimental setup was demonstrated by reproducing reference isotherms as part of two National Institute of Standards and Technology (NIST) interlaboratory studies on CO_2_/ZSM-5 (RM8852) and CH_4_/Zeolite-Y (RM8850),^32,34^ as shown in Figure S2 in the Supporting Information. The experimental results of adsorption experiments are reported in terms of the molar net (or excess) adsorption per unit mass of adsorbent:
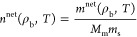
5
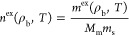
6where *M*_m_ [g mol^–1^] is the molar mass of the sorbate (CO_2_, N_2_, or H_2_).

For the purposes of isotherm modeling, we convert the measured excess amount adsorbed to the absolute amount adsorbed by assuming a constant volume of the adsorbed phase. The latter was taken to be the micropore volume obtained from low pressure N_2_ measurements at 77 K for the three sorbates^35,36^ resulting in
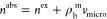
7where ρ_b_^m^ is the molar bulk density of the adsorbate expressed in mol m^–3^, and *v*_micro_ is the micropore volume in units of m^3^ g^–1^. Other methods for determining absolute adsorption such as graphical interpretation of high-pressure excess isotherms, assuming constant density of adsorbed phase,^37^ and other methods^38^ have been described elsewhere.

The values of uncertainty on all experimentally obtained data and parameters were estimated using the general formula for error propagation and details of the procedure have been extensively discussed in our previous works.^24,27^

### 2.4 Equilibrium Isotherm Modeling

#### 2.4.1 Model Descriptions

We model the unary adsorption isotherms using the simplified statistical isotherm (SSI) model first developed by Ruthven^23,39,40^ for zeolitic systems. Zeolites exhibit structurally regular noninteracting cavities within which adsorption is described by pore-filling, rather than surface coverage. The simplified statistical model describes the filling of a subsystem (defined as a supercage for zeolites) of fixed volume *v*_c_ [Å^3^] by one or more adsorbate molecules with an effective molecular volume of β [Å^3^]. The adsorbate–adsorbent interactions is independent of adsorbate concentration but dependent on temperature and, in this work, is modeled by the Henry’s law affinity parameter *K* [molec·supercage^–1^ bar^–1^]. The latter is given by an Arrhenius expression (with constants *K*_0_ [molec·supercage^–1^ bar^–1^] and −Δ*E*_ads_ [kJ mol^–1^]):
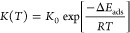
8where *R* is the ideal gas constant [kJ mol^–1^ K^–1^]. The adsorbate–adsorbate interactions are described by the reduction of free volume within the subsystem—the maximum number of adsorbate molecules that can occupy the cage being ω [molec·supercage^–1^]. Given these constraints, the model defines the absolute amount adsorbed in units of molecules per supercage *n*_sc_^abs^ as a function of pressure *p* [bar] and temperature *T* [K] as
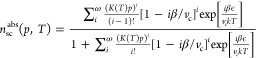
9where *k* is the Boltzmann constant [kJ K^–1^] and ϵ [kJ mol^–1^] is a constant in the exponential factor that accounts for adsorbate–adsorbate intermolecular attractions. In this work, we follow the simplification proposed by Ruthven,^39^ whereby these interactions are neglected (ϵ ≈ 0), yielding:
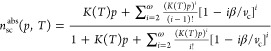
10

To describe experimentally obtained data using this model, a unit conversion is required from the physical units of mol kg^–1^ to molec·supercage^–1^, by using the total volume of cages per unit mass of material, and the volume of an individual cage:
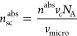
11where *N*_A_ is the Avogadro constant. This conversion assumes that adsorption occurs exclusively in the zeolite cages, and the total volume of these cages corresponds to the micropore volume, *v*_micro_ [Å^3^ kg^–1^]. The latter is obtained by low-pressure adsorption experiments using N_2_ at 77 K (section 2.2). Given the similarity in unit cell structures of X and Y zeolites,^41^ the volume of a single cage was obtained using the unit cell parameters for X zeolites reported by Breck and Grose,^36,42^ yielding *v*_*c*_ = 958.2 Å^3^.

In eq 10, the three unknown parameters are β, *K*_0_, and Δ*E*_ads_, and they are obtained by fitting. These parameters depend on the adsorbate–adsorbent pair, but not on the temperature. In this study, the value of the saturation capacity ω was estimated by using the van der Waals covolume (β_vdW_) for the three gases: 70.9 Å^3^, 64.3 Å^3^, and 44.2 Å^3^ per molecule for CO_2_, N_2_, and H_2_ respectively, along with the cage volume for the zeolite *v*_c_, *i.e*., ω ≈ *v*_c_/β_vdW_ yielding values of 14 (CO_2_), 15 (N_2_), and 22 (H_2_).

We compare this model with the commonly used single-site Langmuir (SSL) model.^43^ The model is given by
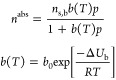
12where *n*^abs^ [mol kg^–1^] is the absolute amount adsorbed, *p* [bar] is the absolute pressure, *n*_s,b_ [mol kg^–1^] is the saturation capacity (fixed for all sorbates for a given material for thermodynamic consistency and obtained by fitting the CO_2_ isotherms first), *b* [bar^–1^] is the temperature dependent adsorption coefficient, described by an Arrhenius expression with two constants, *b*_0_ [bar^–1^] and −Δ*U*_b_ [kJ mol^–1^]. We fit the experimental data to the SSL model to obtain the three fitted parameters, *i.e*., *n*_s,b_, *b*_0_, and −Δ*U*_b_ for CO_2_, and two parameters each, *b*_0_ and −Δ*U*_b_, for both N_2_ and H_2_.

#### 2.4.2 Parameter Estimation and Uncertainty Analysis

We fit the two models to the absolute adsorption isotherms using a maximum likelihood estimator (MLE).^24^ The objective function for the MLE is given as follows:
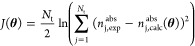
13where *N*_t_ is the total number of data points for pure component *i* at all temperatures, and *n*_j,calc_^abs^(**θ**) is the calculated value of the absolute amount adsorbed at the same *p* and *T* for a set of isotherm parameters given by the vector **θ**. The objective function is minimized using the built-in MATLAB function globalsearch, an algorithm that repeatedly runs a local solver within the bounds set for the parameters, to identify the global optimal values of the respective parameter vectors **θ** for the two models. The resulting vector of optimal parameters is given by θ*.

Assuming the error of the model prediction with respect to the experimental data is normally distributed, the uncertainty bounds at 95% confidence for the estimated parameters were determined by approximating the covariance matrix of the estimated parameter vector θ* as follows:^44^
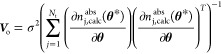
14where σ is the standard deviation of the model with respect to the experimental data given by
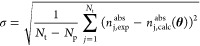
15Here, *N*_p_ is the number of parameters in θ*. ∂*n*_j,calc_^abs^(θ*)/∂**θ** is the sensitivity matrix of the model predicted adsorbed amount with respect to the parameter vector **θ**. Using this, the independent confidence intervals for θ* can be obtained at a probability η, as follows:
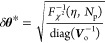
16Here, *F*_χ^2^_^–1^(η, *N*_p_) is the inverse of the chi-squared cumulative distribution function with *N*_p_ degrees of freedom, evaluated at the probability η = 0.95 obtained using the MATLAB function chi2inv. The fitting and uncertainty analysis were implemented using an in-house software package developed using MATLAB R2022a (The Mathworks, Inc.).

## 3 Results and Discussion

### 3.1 As-Prepared and Activated Zeolite NaTMA–Y

**Figure 1:**
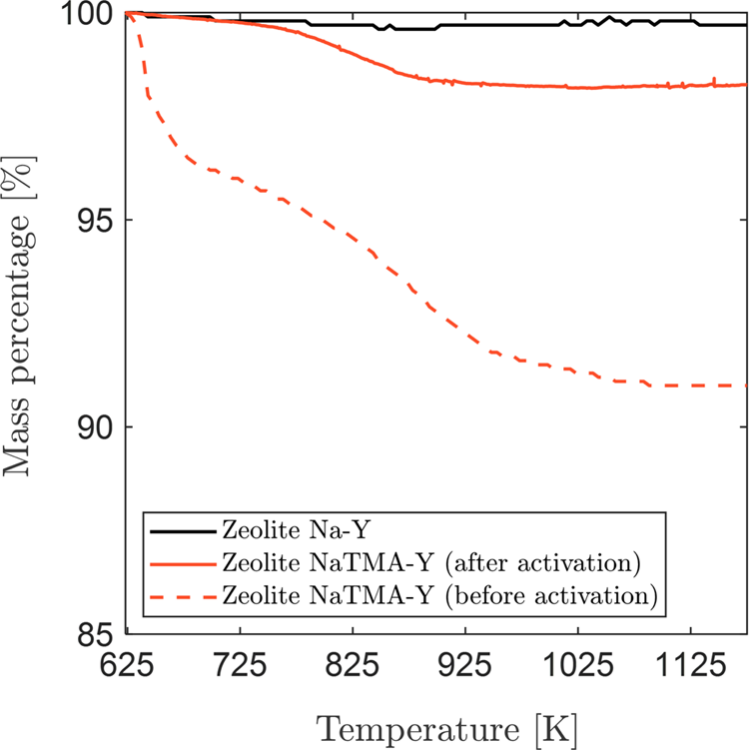
Thermogravimetric analysis was carried out under constant airflow for Zeolite Na–Y and NaTMA–Y (before and after activation). The reduction in mass in this temperature range corresponds to the decomposition of the organic species.

In the following, we quantify the molar cation content of Na^+^ and TMA^+^ in Zeolite Y prior to and after activation. Thermogravimetric analysis (Figure 1) under constant airflow for Zeolite NaTMA–Y before and after activation show a characteristic mass loss between 625 and 1050 K when compared with Zeolite Na–Y, resulting from the thermal decomposition of TMA^+^.^45^ In the Zeolite NaTMA–Y sample before activation, the recorded 8.8% mass loss corresponds to 26.2% molar exchange of Na^+^ to TMA^+^ (the initial Na_2_O content is 13% by mass, as quoted by the manufacturer). As such, we estimate the molar cation content of the as-prepared sample to be 3.1 mol kg^–1^ Na^+^ and 1.1 mol kg^–1^ TMA^+^ (Table 2). This extent of exchange is slightly lower than the value of 31% reported in the literature for the same zeolite.^19^ The incomplete exchange of large cations, such as TMA^+^, can be traced back to steric restrictions to the movement of large cations in and out of the zeolite cages, as discussed extensively in the work of Barrer et al.^46^ In fact, X and Y zeolites contain supercages and β-cages which can be occupied by cations^47^ with aperture free diameters of 7.4 and 2.2 Å respectively.^41^ When compared to the ionic radii of Na^+^ and TMA^+^ (Table 2), it can be inferred that the kinetics of ion exchange between Na^+^ within the β-cages and TMA^+^ in solution may be sterically hindered. Similar conclusions were drawn for TMA^+^ exchange in a ZSM-5 framework by heating in air.^48^ Most notably, as indicated by the TG curves for Zeolite NaTMA–Y, the TMA^+^ content of our sample after activation at 623 K under vacuum for 16 h substantially decreased, yielding 0.2 mol kg^–1^ of TMA^+^ (Table 2). At these conditions, TMA^+^ may already undergo partial decomposition to C–H and N–H compounds, leaving H^+^ within the cages to balance the charge. The TMA-exchanged zeolite Na–Y used for gas adsorption experiments after activation (NaTMA–Y) is thus a partially exchanged sample with a reduced Na^+^ content relative to Na–Y.

**Table 2:** Ionic Diameter of Na^+^ and TMA^+^ along with the Molar Cation Content in H–Y, Na–Y, and NaTMA–Y before and after Activation at 623 K under a Vacuum for 16 h

		molar cation content [mol_ion_ kg^–1^]			
				NaTMA–Y	
cation	*d*_ion_ [Å]	H–Y	Na–Y	before activation	after activation
Na^+^	1.94	0.9	4.2	3.1	3.1
TMA^+^	6.44	0	0	1.1	0.2

### 3.2 Textural Characterization

The textural characterization involved the measurement of skeletal density and pore volume distribution for the three zeolite samples used in the adsorption experiments. The skeletal density was measured by helium gravimetry, as described in section 2.3. Figure S3 in the Supporting Information shows the gravimetric helium isotherms at 393 K for the 3 samples plotted in the form of normalized weight as a function of measured bulk density of helium. For a nonadsorbing inert system, this result is used to determine the volume of adsorbent. Table 3 shows the resulting values of skeletal density (ρ_s_) for the three samples along with the corresponding experimental uncertainty, compared with literature data where applicable.

**Table 3:** Skeletal Density of Zeolites H–Y, Na–Y, and NaTMA–Y Compared to Literature Data Where Available. The Values in Parentheses Represent the Uncertainty Values

			skeletal density	mass of sample
material	reference	remark	[kg m^–3^]	[g]
H–Y	this study	He gravimetry	2130 (80)	0.9464
	Zafar et al.^49^	He pycnometry	2590 (43)	-
Na–Y	this study	He gravimetry	2410 (90)	1.0294
	Nguyen et al.^32^	Data set 2(a)	2480	-
		Data set 2(b)	2490	-
		Data set 3	2040 (17)	-
		Data set 8	2370	-
		Data set 15	2290	-
		Data set 26	2530	-
	Nguyen et al.^50^	He pycnometry	2523 (12)	-
	Verboekend et al.^51^	He pycnometry	2300	-
NaTMA–Y	this study	He gravimetry	2310 (100)	0.8431

The skeletal densities measured in this work differ from the literature for both H–Y and Na–Y. We note that the literature data for Na–Y tend to vary noticeably, too. These differences can be due to the fact that helium adsorbs within micropores, negating the nonadsorbing assumption made in the calculation of the skeletal density.^52−55^ To minimize this issue, helium isotherms for zeolites should ideally be measured at the regeneration temperature (623 K for these samples) across the pressure range used for the equilibrium measurements.^56^ In this study, we were restricted to a maximum temperature of 393 K due to limitations on the experimental setup arising from the use of an external circulating thermostat for temperature control within the sample cell during equilibrium measurements. The reference value for ρ_s_ of Na–Y was reported to be 2523 ± 12 kg m^–3^ as part of the findings from NIST.^50^ For this study, we measured and used a value of 2410 kg m^–3^ to compute the excess adsorbed amount using eq 6. We compare the excess isotherms for Na–Y computed using the two different values for ρ_s_, and we show the relative deviation between the two sets of data in Figure S5 in the Supporting Information. The comparison shows maximum deviations of 0.35%, 1.65%, and 6.71% in *n*^ex^ for CO_2_, N_2_, and H_2_, respectively. As in our previous work, we report the net adsorption for all measured isotherms along with the excess and absolute amounts, which can be used to compute the excess adsorption using any value for ρ_s_.

Table 4 summarizes the BET area, micropore volume, and microporosity obtained from the NLDFT model calculations on the N_2_ physisorption isotherms measured at 77 K. These isotherms and the resulting pore-size distributions for cumulative and differential volume for the three samples are shown in Figure S4 in the Supporting Information. All three isotherms are Type-I^57^ and show no hysteresis, as expected for rigid microporous materials.^50^ Both Na–Y and NaTMA–Y are primarily microporous, the latter showing a reduced BET area and micropore volume relative to Na–Y. H–Y displays some mesoporosity; the mechanism for the appearance of mesoporosity has been proposed to be the formation of cavities in the crystals upon dealumination of the zeolite during production. The dealumination process in the production of H–Y also causes confinement of aluminum ions within zeolite unit cells, thereby reducing the micropore volume.^58^ The micropore volume for H–Y and Na–Y agree with the reported values in the literature (0.256 cm^3^ g^–1^ for H–Y^58^ and 0.358 cm^3^ g^–1^ for Na–Y^50^). The calculated BET areas for H–Y and Na–Y shown in Table 4 are also in good agreement with values reported by the manufacturer (730 m^2^ g^–1^ for H–Y and 900 m^2^ g^–1^ for Na–Y).^59^

**Table 4:** Textural Properties of the Adsorbent Materials Derived from N_2_ Adsorption Isotherms at 77 K

	BET area	micropore volume	microporosity
material	[m^2^ g^–1^]	[cm^3^ g^–1^]	[%]
H–Y	741	0.260	74
Na–Y	914	0.359	94
NaTMA–Y	883	0.344	94

### 3.3 CO_2_, N_2_, and H_2_ Excess Adsorption Isotherms

**Figure 2:**
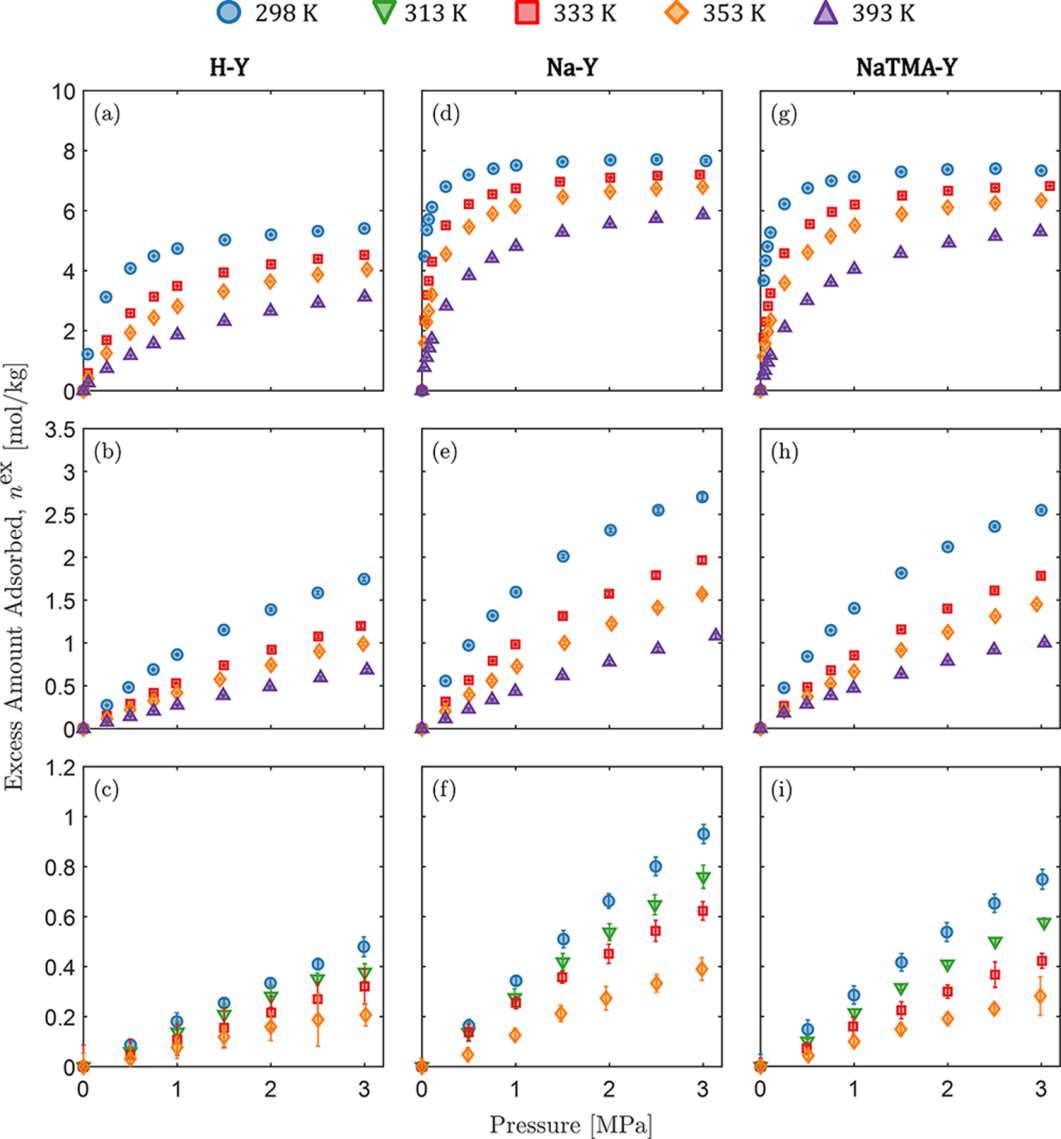
Excess adsorption isotherms of (a, d, g) CO_2_, (b, e, h) N_2_, and (c, f, (i) H_2_ on Zeolite H–Y (a-c), Na–Y (d-f), and NaTMA–Y (g-i), at various temperatures. The error bars correspond to one standard deviation of the measured quantity and are computed using the general formula for error propagation.

The unary excess adsorption isotherms for CO_2_, N_2_, (298.15 K, 313.15 K, 333.15 K, 353.15 K, 393.15 K) and H_2_ (298.15 K, 313.15 K, 333.15 K, 353.15 K) are presented in Figure 2 along with the corresponding error bars (one standard deviation). The data and their uncertainties are tabulated in Table S2 in the Supporting Information together with the corresponding net amount adsorbed, from which they have been estimated. For each zeolite sample, the adsorption capacity decreases with temperature and in the following order: CO_2_ > N_2_ > H_2_. In the pressure range tested, the isotherms measured with CO_2_ show the strongest degree of nonlinearity, followed by N_2_ and H_2_. For the latter, the measured isotherms are by and large linear at all temperatures. When comparing the three samples, the adsorption capacity decreases across all gases for the three zeolites in the order: Na–Y ≥ NaTMA–Y > H–Y. The similarity between the isotherms measured on Na–Y and NaTMA–Y is largely due to the limited cation exchange achieved (Table 2) and the similar micropore volume of these two samples (Table 4). The relative uncertainties in the measurements for CO_2_ and N_2_ are significantly smaller compared to that for H_2_. This was also observed in our previous work and is attributed to the low observed weight change during adsorption due to the coupled effects of inherently low adsorption capacity and low molecular weight of H_2_.^24^ The uncertainties in all measurements fall within the same order of magnitude when expressed in units of g g^–1^ (Table S2 in the Supporting Information).

### 3.4 Absolute Adsorption and Simplified Statistical Isotherm Model

**Figure 3:**
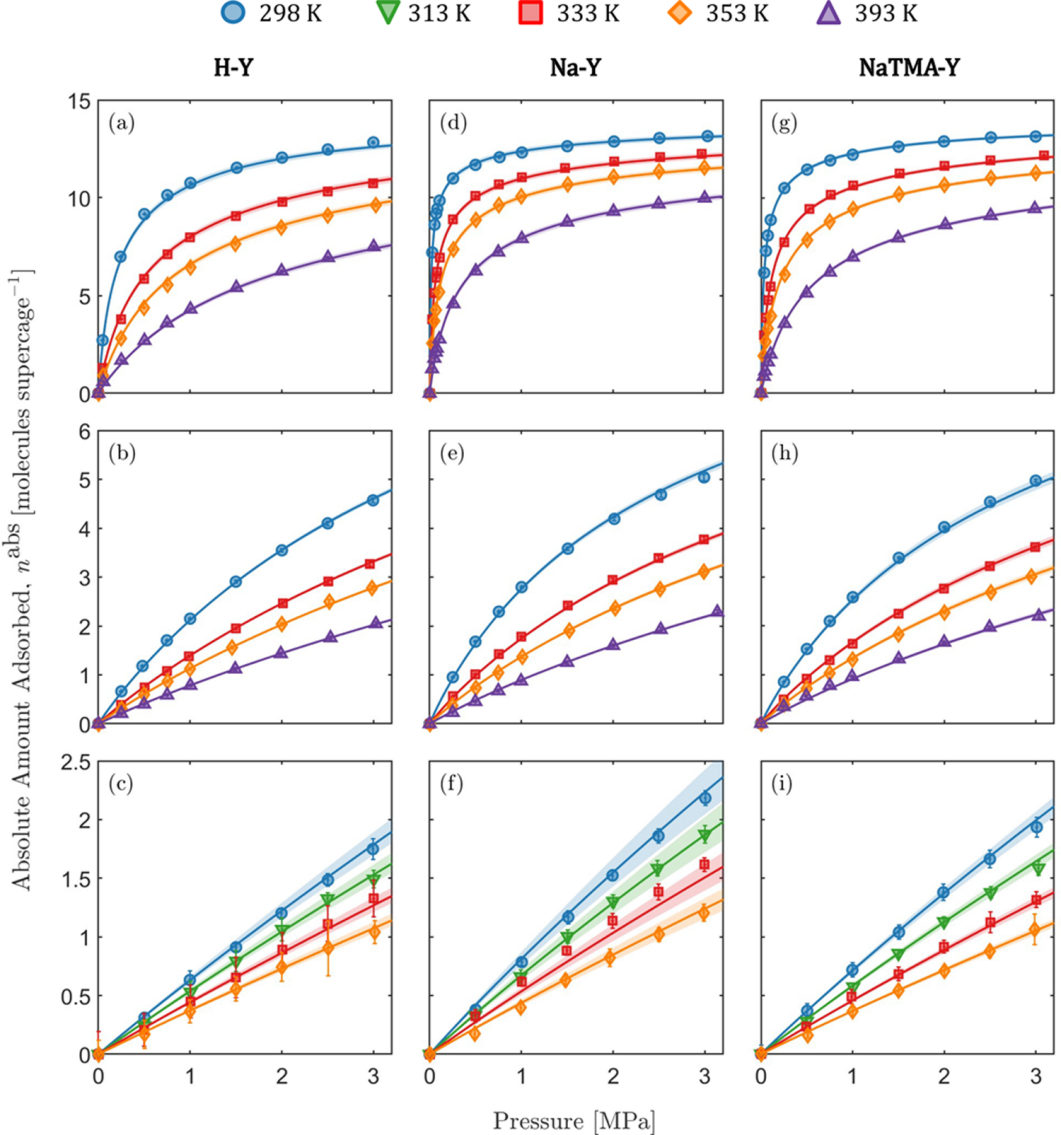
Absolute adsorption isotherms of (a, d, g) CO_2_, (b, e, h) N_2_, and (c, f, (i) H_2_ on Zeolite H–Y (a-c), Na–Y (d-f), and NaTMA–Y (g-i), at various temperatures in units of molecules/supercage. The solid lines represent the isotherm fitting to the simplified statistical isotherm (SSI) model given by eq 10, and the shaded regions show 95% confidence bounds.

To analyze the experimental data, the excess isotherms were converted to absolute isotherms by using eq 7. To discuss particular trends observed in the experimental data and link them to the structure and chemistry of the zeolites, the experimental data were further converted from units of mol kg^–1^ to molecules per supercage by using eq 11. Following this, the absolute isotherm data was fitted to the simplified statistical isotherm (SSI) model, eq 10, as shown in Figure 3. The resulting fitted parameters along with their uncertainties are listed in Table 5. The same model fits to the absolute isotherms in units of mol kg^–1^ are shown in Figure S6 in the Supporting Information.

**Table 5:** Simplified Statistical Isotherm (SSI) Model Parameters Derived from Fitting the CO_2_, N_2_, and H_2_ Isotherms. The Values in Parentheses Represent the Uncertainty Values Where Available. The Values of Parameter ω Are Fixed and Not Obtained by Fitting

	Simplified Statistical Isotherm (SSI)				
	ω		β	*K*_0_×10^4^	–Δ*E*_ads_
	[molec ·supercage^–1^]	[mol kg^–1^]	[Å^3^]	[molec·supercage^–1^ bar^–1^]	[kJ mol^–1^]
	H–Y				
CO_2_	14	6.31	47.52 (0.65)	5.22 (0.14)	23.77 (0.08)
N_2_	15	6.76	46.82 (0.75)	23.56 (0.14)	11.87 (0.02)
H_2_	22	9.91	20.18 (5.78)	20.06 (0.43)	8.66 (0.06)
	Na–Y				
CO_2_	14	8.71	57.26 (0.60)	1.77 (0.06)	32.91 (0.10)
N_2_	15	9.33	58.83 (1.16)	12.40 (0.13)	14.57 (0.03)
H_2_	22	13.69	27.22 (8.38)	16.13 (0.53)	9.91 (0.09)
	NaTMA–Y				
CO_2_	14	8.34	54.62 (0.47)	1.84 (0.04)	31.07 (0.07)
N_2_	15	8.94	58.80 (1.78)	20.01 (0.33)	13.08 (0.05)
H_2_	22	13.12	22.35 (4.44)	9.04 (0.14)	10.96 (0.04)

As shown in Figure 3, the SSI model provides an excellent fit of the experimental data at all temperatures for each gas and for each sample. Notably in the units of molecules per supercage, the three adsorbents show similar adsorption capacity for each gas, indicating that the differences observed when the same data are reported per unit mass of adsorbent (Figure S6 in the Supporting Information) are due to the difference in the number of cages per unit mass of zeolite crystal, which is reflective of differences in the micropore volume, (*v*_micro_/*v*_*c*_). Because the adsorbents are all Y zeolites with FAU unit cells and exhibit the same Si/Al ratio (Si:Al = 2.55), their comparison in these units can shed light on the effect of the cation on the adsorption of molecules within a single cage.

In the following, we discuss the obtained model parameter values and their relationship with the thermodynamics of adsorbate interaction with the zeolites. The parameter ω represents the saturation capacity for a single cavity or supercage. In our study, this parameter was not fitted; rather, we estimated it by using the van der Waals covolume β_vdW_ and the cage volume for the zeolites *v*_c_ as described in section 2.4.1. As such, for each gas, ω takes the same value for the three adsorbents. The ability of the SSI model to accurately describe the isotherms further implies that the cation has a negligible effect on the saturation capacity of the adsorbent. The effective molecular volume of the adsorbed molecules (β) was obtained by fitting and can be compared with van der Waal’s covolume (β_vdW_) for each sorbate: 70.9 Å^3^, 64.3 Å^3^, and 44.2 Å^3^ per molecule for CO_2_, N_2_, and H_2_, respectively. The fitted values for β (Table 5) are smaller than the van der Waal’s covolume and show slight variations between adsorbents. The reduction in the effective molecular volume of adsorbed species can be attributed to compressibility effects at high pressures. Notably, the effective molecular volumes for adsorbed CO_2_ and N_2_ on all three zeolites are similar to those predicted in previous works.^36,39^ However, as with the experimental data, the uncertainty in β is relatively high for H_2_ compared to the other adsorbates and we attribute this to the uncertainty in the experimental data (as is reflected in the confidence bounds shown as shaded regions in Figure 3).

### 3.5 Adsorption in the Henry’s Law Region

**Figure 4:**
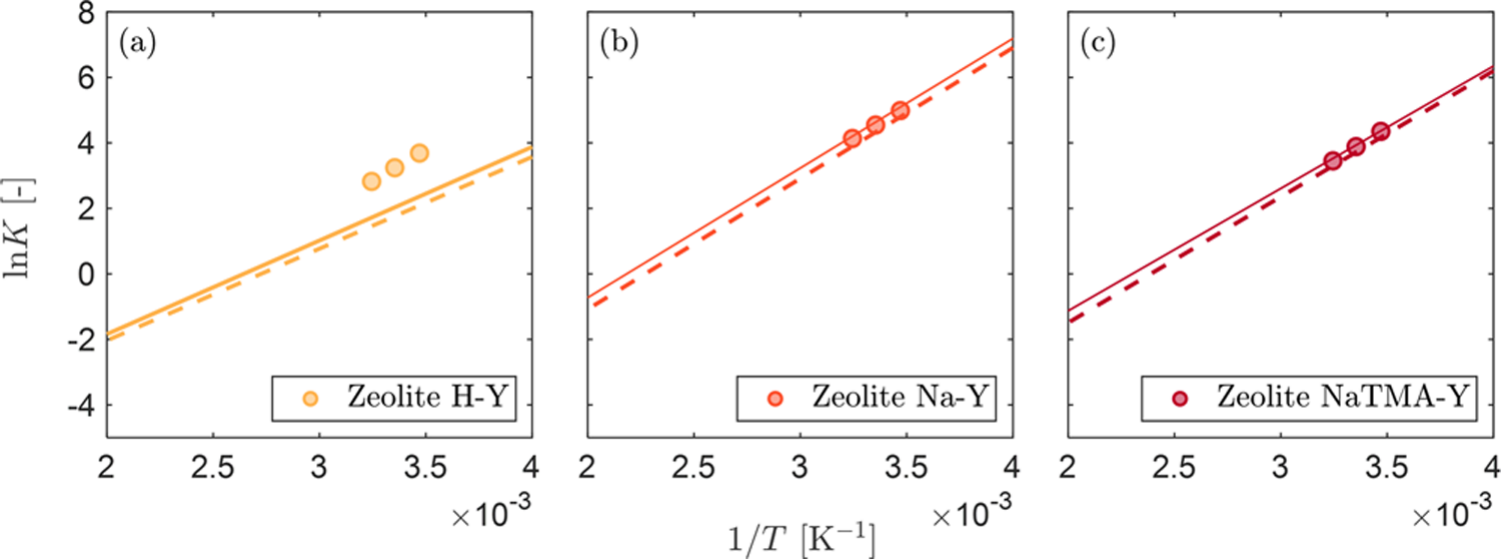
van’t Hoff plot of the natural logarithm of the Henry’s constant *K* for CO_2_ predicted by the simplified statistical isotherm (SSI) model (solid lines) and single-site Langmuir (SSL) model (dashed lines) compared with the values obtained from low pressure experimental data at 288, 298, and 309 K on (a) Zeolite H–Y, (b) Na–Y, and (c) NaTMA–Y (circles).

When applied to zeolites, eq 10 provides a useful method of extracting the Henry’s law constants (*K*_0_ and Δ*E*_ads_) using experimental data obtained outside the Henry’s law region. Accurate prediction of Henry’s law behavior is important when the performance of the adsorbent is determined by the equilibrium data at low pressures, *e.g*., postcombustion carbon capture applications.^6,7^ To demonstrate the accuracy of the predicted Henry’s law constants for the three zeolites, we produced a van’t Hoff plot (ln *K* vs 1/*T*), comparing the values predicted by the SSI and SSL models for CO_2_ with those obtained directly from isotherm measurements at 288 K, 298 K and 309 K (Figure 4). The latter were calculated by fitting the data to the virial isotherm model using the methodology described in section S10 of the Supporting Information. The results show excellent agreement between the model and the experimental data for Na–Y and NaTMA–Y, but an underprediction of *K* for H–Y. We attribute this to the sparsity of data points below 0.1 MPa for H–Y (2 points) compared to Na–Y and NaTMA–Y (5 points) in the gravimetric measurements.

The Henry’s law constants (*K*) obtained from the SSI model for the experimentally measured isotherms are reported in Table 6. For each gas, the Henry’s constants decrease in the order Na–Y > NaTMA–Y > H–Y. The observed trend correlates with the strength of adsorbate–adsorbent interaction and, more precisely, the effect of the cation on the affinity of each adsorbate. The comparison of CO_2_ adsorption on Na–Y and NaTMA–Y is particularly noteworthy as both materials have a similar CO_2_ capacity at 3 MPa while *K* for NaTMA–Y is approximately half of that for Na–Y throughout the temperature range. This indicates weaker interaction between CO_2_ and the NaTMA–Y framework at low coverage while maintaining a high capacity at high pressures. This can be exploited in applications such as precombustion carbon capture using a pressure-swing process where the feed partial pressure of CO_2_ is relatively high.^5^ H–Y on the other hand exhibits the lowest *K* values which can be attributed to the lower basicity of the framework resulting from the low alkali metal content.^60^

**Table 6:** Henry Constants (*K*) Determined from the Simplified Statistical Isotherm Parameters: *K*_0_ and Δ*E*_ads_. The Values in Parentheses Represent the Uncertainty Values

	*K*		
*T*	[molec·supercage^–1^ bar^–1^]		
[K]	H–Y	Na–Y	NaTMA–Y
CO_2_			
298.15	7.63 (0.201)	102.97 (3.266)	51.04 (1.167)
333.15	2.79 (0.074)	25.53 (0.810)	13.68 (0.313)
353.15	1.71 (0.045)	13.02 (0.413)	7.25 (0.166)
393.15	0.75 (0.020)	4.16 (0.132)	2.47 (0.057)
N_2_			
298.15	0.28 (0.0017)	0.44 (0.0046)	0.39 (0.0065)
333.15	0.17 (0.0010)	0.24 (0.0025)	0.23 (0.0037)
353.15	0.13 (0.0008)	0.18 (0.0018)	0.17 (0.0029)
393.15	0.09 (0.0005)	0.11 (0.0011)	0.11 (0.0018)
H_2_			
298.15	0.066 (0.0014)	0.088 (0.0029)	0.075 (0.0011)
313.15	0.056 (0.0012)	0.073 (0.0024)	0.061 (0.0009)
333.15	0.046 (0.0010)	0.058 (0.0019)	0.047 (0.0007)
353.15	0.038 (0.0008)	0.047 (0.0015)	0.038 (0.0006)

## 4 Perspectives on Measurements

### 4.1 Isotherm Model Comparison

**Figure 5:**
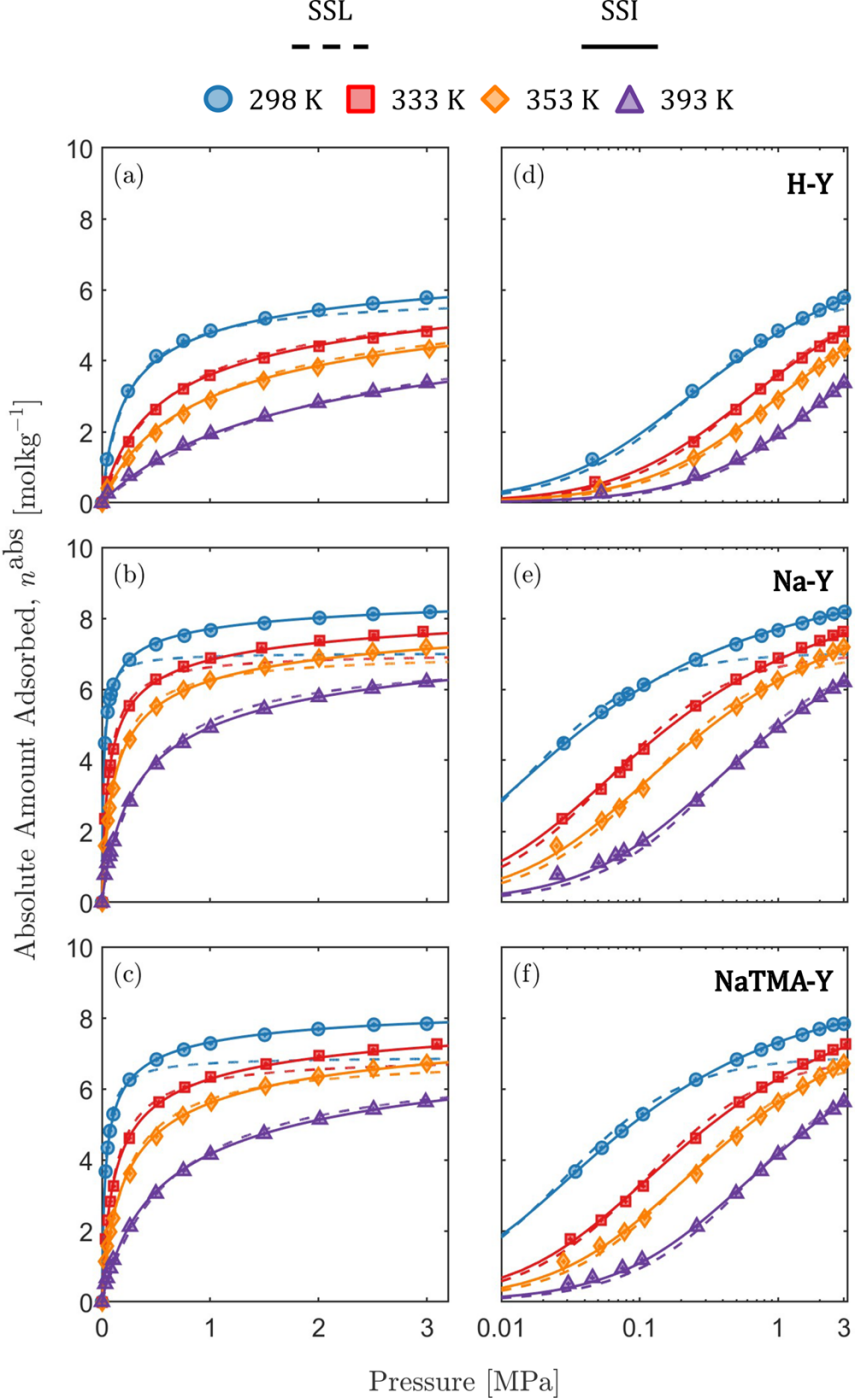
Absolute adsorption isotherms of CO_2_ on Zeolite (a,d) H–Y, (b,e) Na–Y, and (c,f) NaTMA–Y, comparing the single-site Langmuir (dashed lines) and simplified statistical isotherm model (solid lines) shown on linear (left) and logarithmic (right) pressure scales.

The SSL model (eq 12) is commonly used in process modeling as it can be readily extended to a formulation for multicomponent gas mixtures and has been shown to often provide sufficiently accurate description of experimental data with minimum computational complexity.^6,8,61^ However, for zeolite systems, particularly at high pressures or near saturation conditions, the assumptions made in the formulation of this model break down.^36^ We compare the SSI model with the SSL for CO_2_ adsorption in Figure 5 where the two models are presented in units of mol kg^–1^. The comparisons for N_2_ and H_2_ are shown in Figure S8 in the Supporting Information. The relative deviations of the two models with respect to the experimental data are shown in Figures S10, S11, and S12 in the Supporting Information. The SSL isotherm model parameters derived from fitting the CO_2_, N_2_, and H_2_ isotherms for the three zeolites are given in Table 8.

**Table 8:** Single-Site Langmuir Isotherm Model Parameters Derived from Fitting the CO_2_, N_2_, and H_2_ Isotherms. The Values in Parentheses Represent the Uncertainty Values

	Single-Site Langmuir (SSL)		
	*n*_s,b_	*b*_0_×10^7^	–Δ*U*_b_
	[mol kg^–1^]	[bar^–1^]	[kJ mol^–1^]
	H–Y		
CO_2_	5.86 (0.05)	370.44 (15.54)	23.31 (0.12)
N_2_		193.42 (19.60)	11.35 (0.03)
H_2_		1406.86 (28.54)	8.96 (0.05)
	Na–Y		
CO_2_	7.03 (0.14)	102.10 (7.99)	33.21 (0.25)
N_2_		1347.59 (24.63)	13.44 (0.05)
H_2_		1035.24 (30.86)	10.79 (0.08)
	NaTMA–Y		
CO_2_	6.91 (0.12)	88.59 (5.07)	31.98 (0.18)
N_2_		2078.48 (50.90)	12.00 (0.07)
H_2_		744.94 (8.28)	11.16 (0.03)

The SSI model describes the CO_2_ data excellently over the entire pressure range, as discussed previously. Conversely, the SSL model describes the CO_2_ data fairly well for H–Y, and the quality of fit is poor for Na–Y and NaTMA–Y near saturation at high pressures. The SSL model postulates localized adsorption on energetically homogeneous sites with no interaction between adsorbate molecules and can be readily obtained by imposing ω = 1 in the SSI model (eq 10). As discussed previously, this conceptualization of the adsorption process is not suitable for zeolite systems that instead exhibit micropore filling (as opposed to surface coverage), *i.e*., ω > 1 and each “site” (*i.e*., pore) can be occupied by more molecules (up to 14 for CO_2_). Nevertheless, the assumptions of the SSL model are sufficient to provide a relatively good fit for cases where the adsorbate–adsorbent attraction is weaker, as in the cases of CO_2_ on H–Y, and N_2_ and H_2_ on all three zeolites (see Figure S8 in the Supporting Information). Extensions of the Langmuir model, *e.g*., the dual-site Langmuir (DSL) model (consisting of six fitted parameters), have been developed to model systems that cannot be suitably described by the SSL model.^11^ While the increased number of fitting parameters will improve the fit in comparison to the SSL model, the formulation would still lack a physical basis for zeolite systems, and the fitting exercise reduces to a mere mathematical description of experimental data. We have fit the absolute isotherm data to the DSL isotherm model and show the resulting fits compared to the SSI model in Figure S9 in the Supporting Information, with the fitted parameters in Table 8 in the Supporting Information. The results, particularly for CO_2_, show that the DSL model describes the experimental data sufficiently well at low pressures, as has been shown in our previous work on zeolite 13X,^62^ but not at pressures near the saturation conditions. This problem can be countered by adding more parameters to the Langmuir models, *e.g*., incorporating temperature dependence of the saturation capacity of the sorbate in the model. Such approaches may provide a better empirical fit to the data, albeit at the cost of introducing issues with the thermodynamic consistency of the model formulation.

### 4.2 Literature Comparison

**Figure 6:**
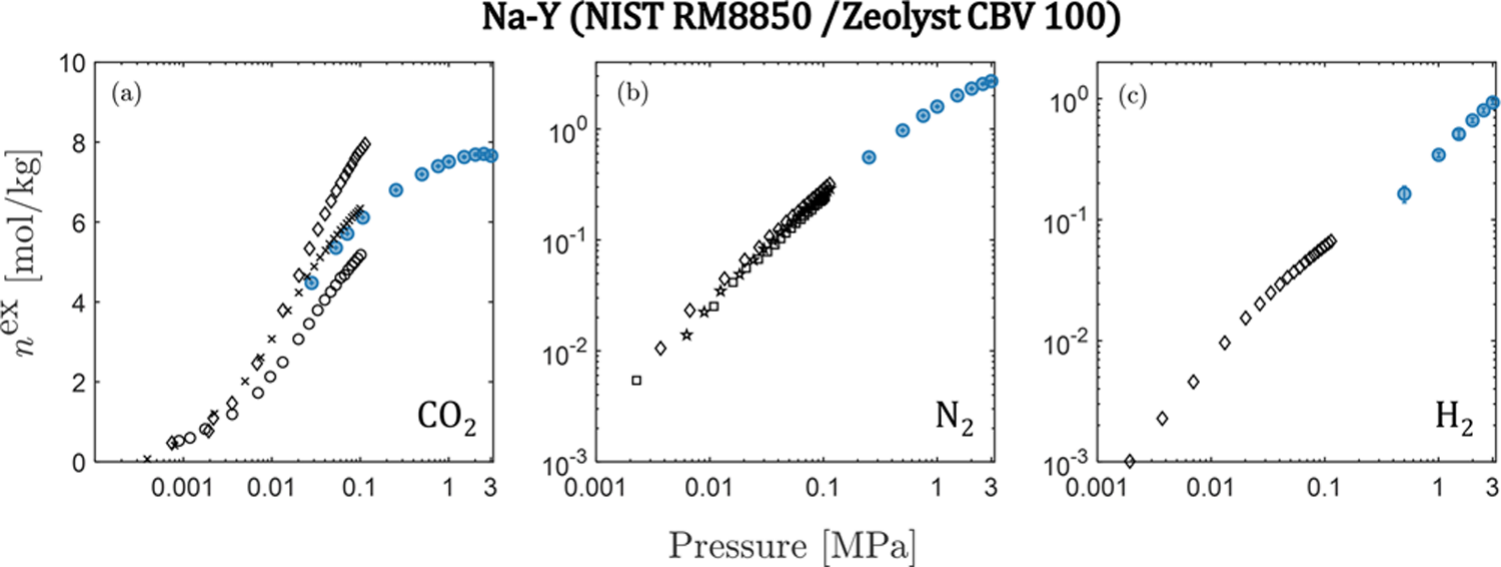
Excess adsorption isotherms on Zeolite Na–Y for (a) CO_2_, (b) N_2_, and (c) H_2_ measured in this work (blue) at 298 K, compared to literature data (black) from Kim et al.^63^ at 298 K (diamonds), Wong-Ng et al.^65^ at 298 K (crosses), Pham et al.^64^ at 303 K (circles), Wu et al.^21^ at 298 K (squares), and Li et al.^19^ at 303 K (pentagrams).

As previously discussed, Y-zeolites have been widely studied for various carbon capture applications. In the following, we compare our data with the literature, where it is available. To this end, we restricted our search for H–Y and Na–Y to the same product by the same manufacturers so as to eliminate bias in the data associated with different chemical composition arising from the synthesis of the zeolites. For the case of H–Y, we were unable to find any adsorption isotherms for the gases studied. Figure 6 shows the comparison of available data for Na–Y (for NIST RM8850 and Zeolyst CBV100) with the data obtained in this study. Our data agree well with that available in the literature, but show some disagreement for CO_2_ when compared to data from Kim et al.^63^ and Pham et al.^64^

**Figure 7:**
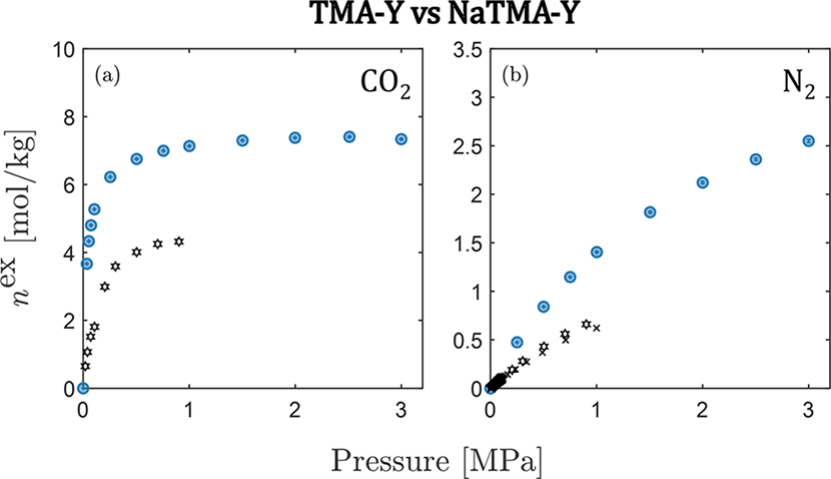
Excess adsorption isotherms on Zeolite NaTMA–Y for (a) CO_2_, and (b) N_2_ measured in this work (blue) at 298 K, compared to literature data for Zeolite TMA–Y (black) from Avijegon^20^ at 303 K (hexagrams), Wu et al.^21^ at 298 K (squares), Hu et al.^66^ (crosses), and Li et al.^19^ (pentagrams).

We also compare the isotherms obtained for CO_2_ and N_2_ on NaTMA–Y with data for TMA–Y produced from the same parent Na–Y as in this work (Figure 7). We observe that TMA–Y has a lower capacity for both CO_2_ and N_2_ compared to NaTMA–Y, which is also consistent with the smaller micropore volume for TMA–Y (0.17 cm^3^ g^–1^ for TMA–Y^22^ vs 0.344 cm^3^ g^–1^ for NaTMA–Y in this work). As anticipated in section 3.1, this difference can be explained by the partial cation exchange achieved in this study.

## 5 Conclusions

We have reported gravimetric measurements of adsorption of CO_2_, N_2_ (298.15 to 393.15 K), and H_2_ (298.15 to 353.15 K) on commercial Zeolites H–Y and Na–Y, and cation-exchanged NaTMA–Y in the pressure range of vacuum to 3 MPa. We presented the excess adsorption isotherms as measured, net adsorption isotherms for use with different values for the skeletal density of the sorbent, along with the absolute adsorption isotherms. Absolute isotherms were computed by assuming a constant adsorbed phase volume for use with analytical and empirical equilibrium isotherm models. We modeled the absolute isotherms for the three gases using a simplified statistical isotherm model which provides an improved description of the equilibrium data close to the saturation capacity for CO_2_ sorption on Na–Y and NaTMA–Y when compared to the commonly used Langmuir model. The Henry’s constants decrease across all gases for the three zeolites in the order Na–Y > NaTMA–Y > H–Y indicating that the adsorbate–adsorbent interactions decrease following the same order. The difference between Na–Y and NaTMA–Y is noteworthy: for a similar CO_2_ adsorption capacity at 3 MPa at a supercage scale, the Henry’s constants are reduced by half for the latter. The textural properties and isotherm model parameters can be used as presented in process simulators to evaluate different adsorption-based processes.

## Data Availability

The software package used for isotherm fitting and uncertainty calculation is available on the Imperial College London main repository on GitHub and can be accessed at https://github.com/ImperialCollegeLondon/IsothermFittingTool.

## Supporting Information Available

Dual-site Langmuir (DSL) model parameters for absolute adsorption of CO_2_, N_2_, and H_2_; virial isotherm model and fitted parameters; additional figures and tables supporting the text (PDF)
Experimental data (ZIP)
