# Supplementary material for: Unary Adsorption Equilibria of Hydrogen, Nitrogen, and Carbon Dioxide on Y-Type Zeolites at Temperatures from 298 to 393 K and at Pressures up to 3 MPa
Source: J Chem Eng Data. 2023 Oct 30;68(12):3512–24. doi: 10.1021/acs.jced.3c00504 (PMC10726315; doi:10.1021/acs.jced.3c00504)
Supplement: Supplementary file 1 — je3c00504_si_001.pdf [file je3c00504_si_001.pdf]

Supporting Information (SI) for "Unary Adsorption Equilibria of Hydrogen, Nitrogen and Carbon Dioxide on Y-type Zeolites at Temperatures from 298 to 393 K and at Pressures up to 3 MPa"

Hassan Azzan,[†] David Danaci,[†] Camille Petit[†] Ronny Pini,[†]

Contents

S1 Bulk gas density measurements **2**

S2 Reference isotherms for Zeolite-Y (RM8850) and ZSM-5 (RM8852) **3**

S3 Gravimetric helium isotherms at 393 K **4**

S4 Volumetric nitrogen isotherms at 77 K **and pore-size distributions** **5**

S5 Excess isotherms for Na-Y using two different values of ρ_s **6**

S6 Absolute isotherms and SSI model fits in units of $\mathrm{mol\,kg^{-1}}$ **7**

S7 Low pressure volumetric CO_2 adsorption isotherms **8**

S8 Comparison of single-site Langmuir (SSL) and SSI models fits for absolute adsorption of N_2 and H_2 **9**

S9 Dual-site Langmuir (DSL) model parameters for absolute adsorption of CO_2, N_2 and H_2 **10**

S10 Comparison of dual-site Langmuir (DSL) and SSI models fits for absolute adsorption of CO_2, N_2 and H_2 **11**

S11 Relative deviation plots for SSL and SSI models **12**

S12 Virial isotherm model and fitted parameters **14**

S13 Excess, net, and absolute isotherm data for H-Y, Na-Y and NaTMA-Y **16**

[*] Email: rpini@imperial.ac.uk

[†] Department of Chemical Engineering, Imperial College London, London, SW7 2AZ, United Kingdom

S1 Bulk gas density measurements

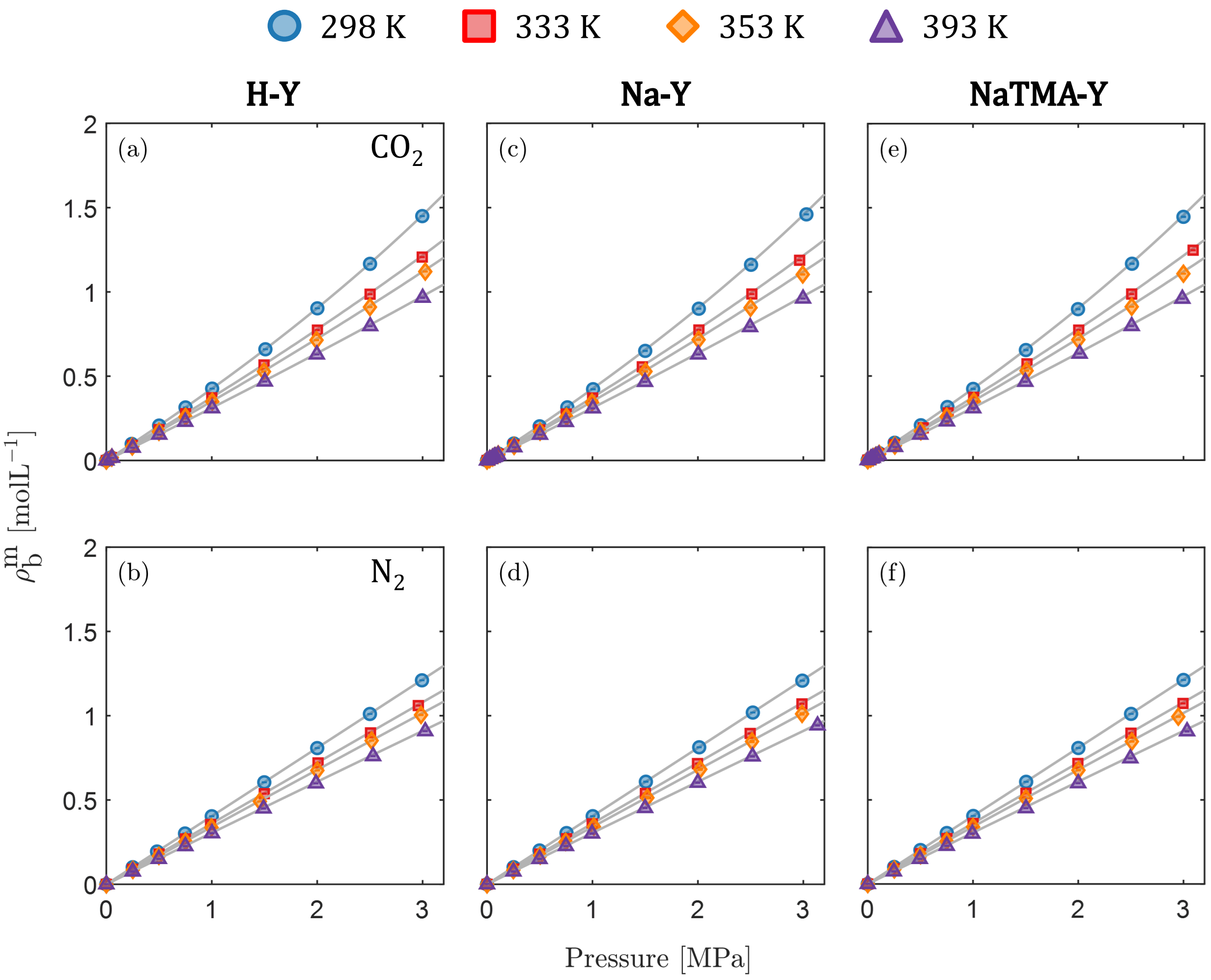

Figure S1: Bulk phase density measured *in situ* by the magnetic suspension balance compared to the values calculated using appropriate equations of state[1] for equilibrium measurements of CO_2 and N_2 on Zeolites (a-b) H-Y, (c-d) Na-Y, (e-f) and NaTMA-Y.

S2 Reference isotherms for Zeolite-Y (RM8850) and ZSM-5 (RM8852)

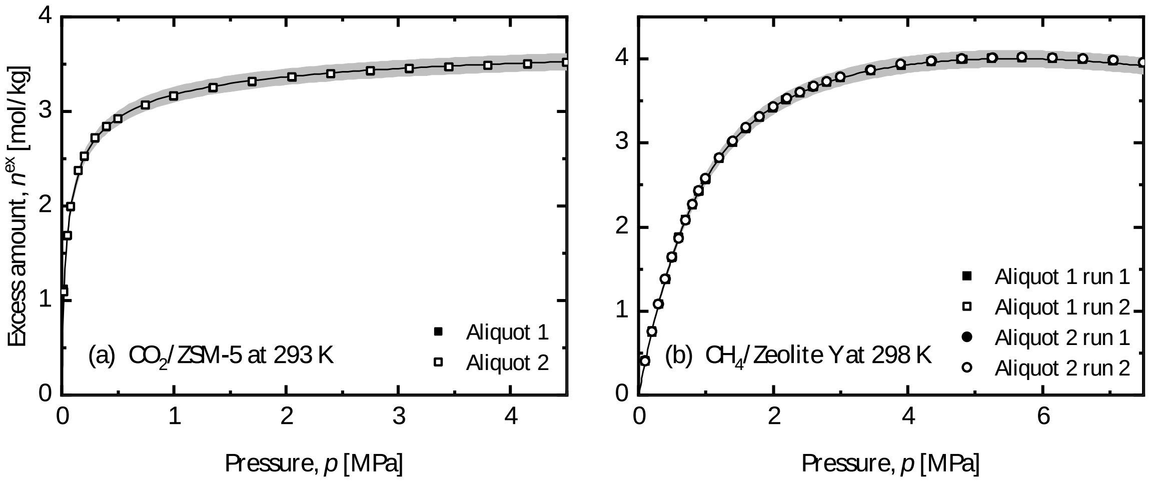

Figure S2: Excess adsorbed amounts of (a) CO_2 on ZSM-5 at 293 K[2] and (b) CH_4 on Zeolite Y at 298 K[3]. The solid lines represent the reference isotherms obtained from an interlaboratory study with the uncertainty bounds represented by the shaded regions.

S3 Gravimetric helium isotherms at 393 K

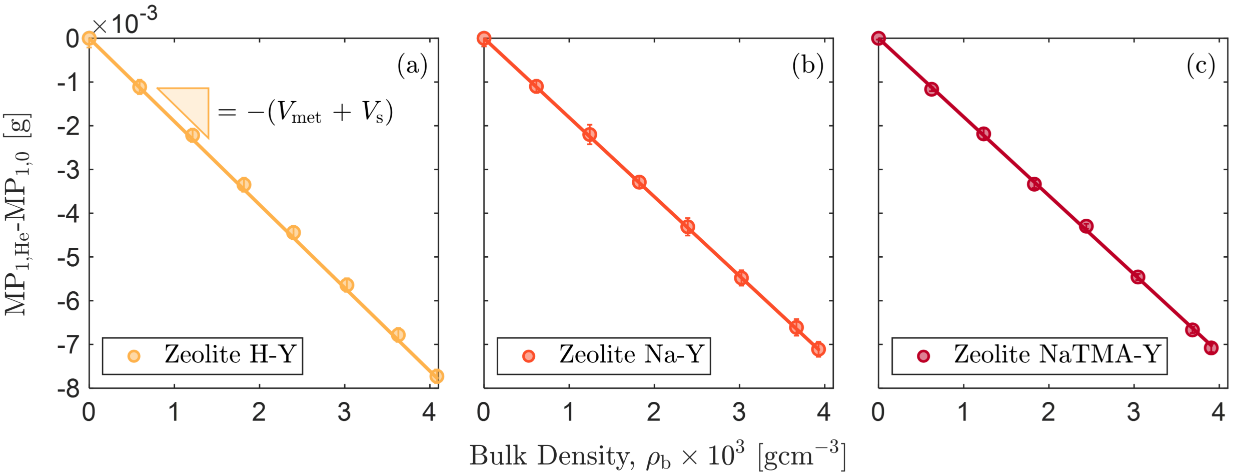

Figure S3: Normalized weight (MP_1–$MP_{1,0}$) plotted against helium bulk density measured at 393 K for (a) Zeolite H-Y, (b) Zeolite Na-Y, and (c) Zeolite NaTMA-Y. The slope of the linear regression line is equivalent to the negative value of $V_0 = V_{met} + V_s$

S4 Volumetric nitrogen isotherms at 77 K and pore-size distributions

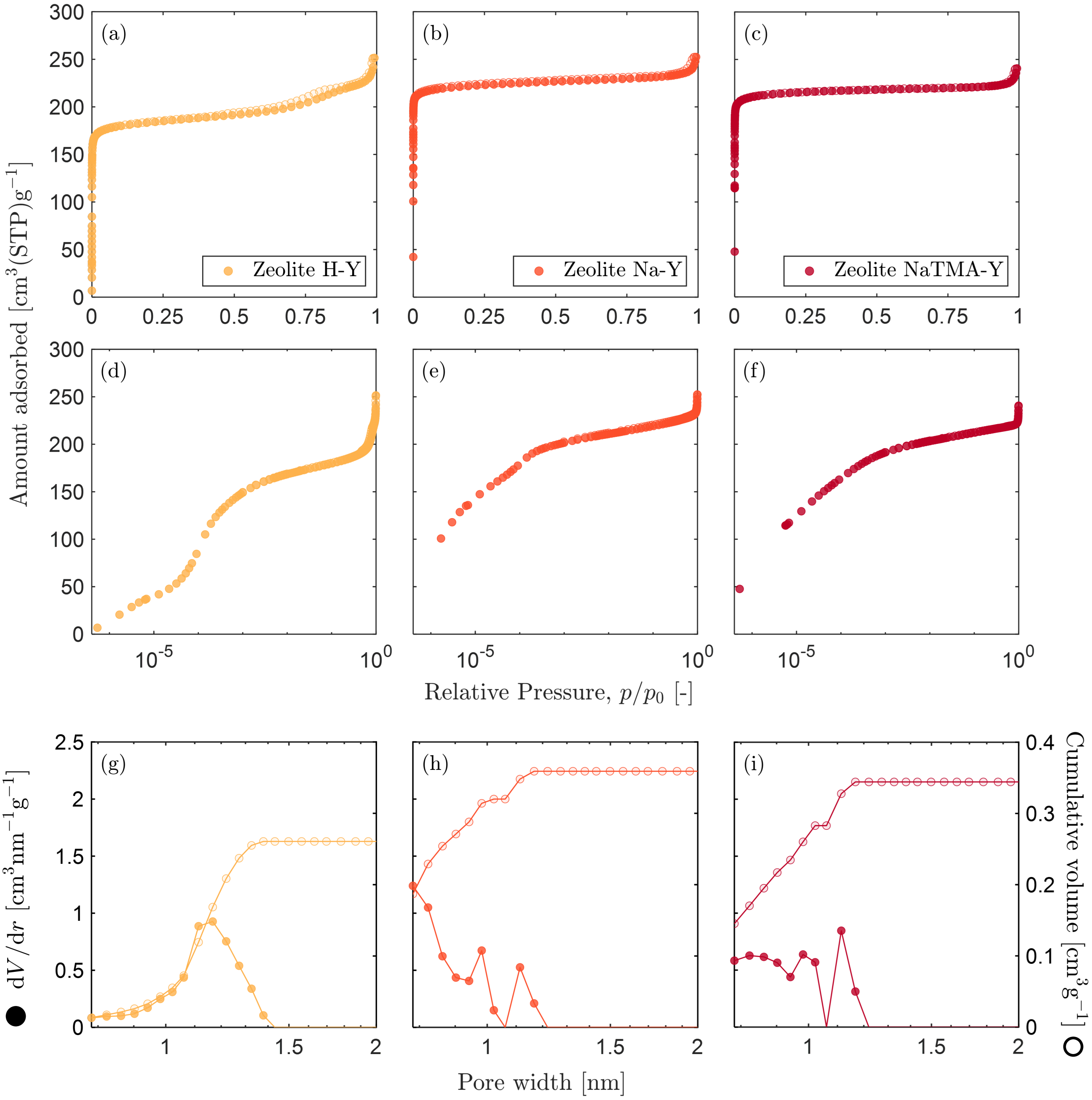

Figure S4: N_2 physisorption (closed symbols) and desorption (open symbols) isotherms on Zeolite H-Y, Na-Y, and NaTMA-Y at 77 K in linear (a-c) and logarithmic relative pressure scales (d-f) and (g-i) cumulative volume (right y-axis) and PSD (left y-axis) obtained from the NLDFT model.

S5 Excess isotherms for Na-Y using two different values of ρ_s

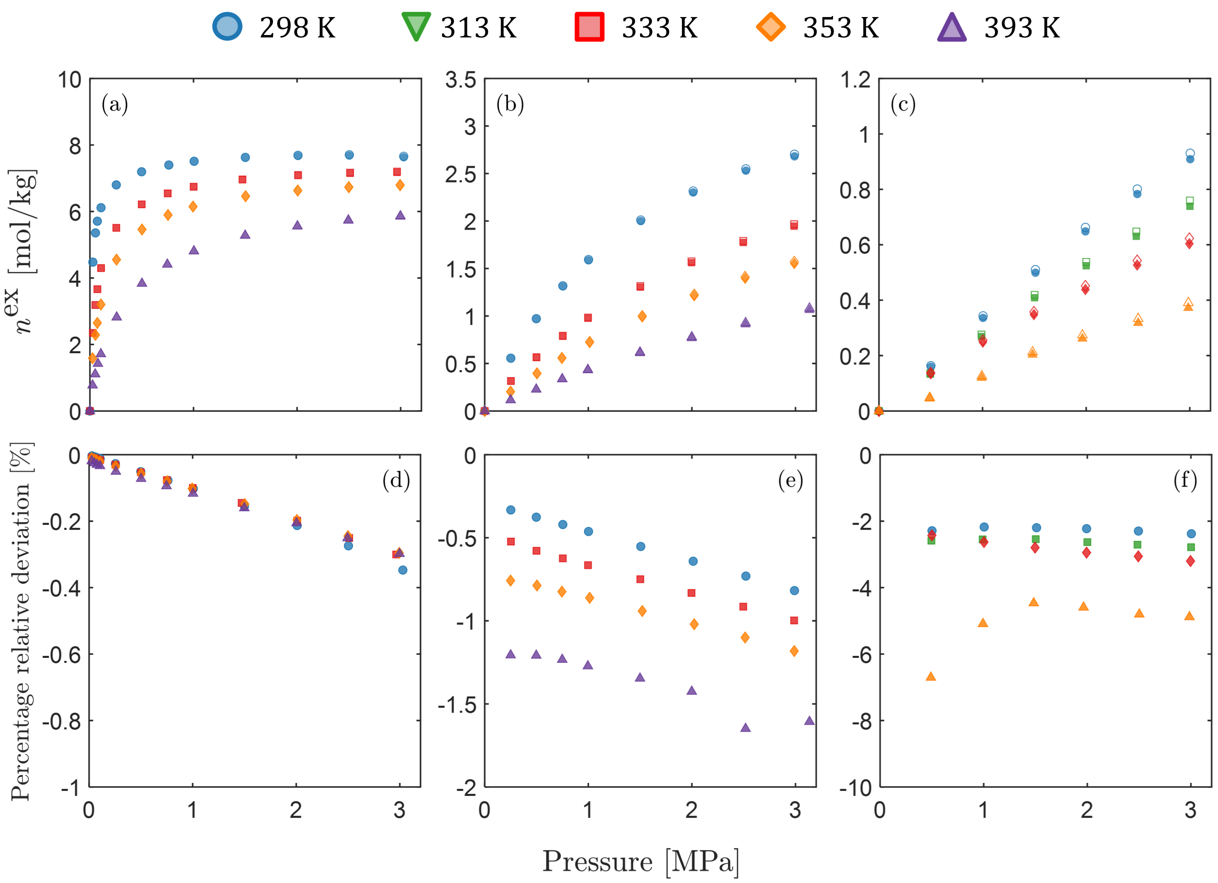

Figure S5: Excess adsorption isotherms of (a) CO_2, (b) N_2, and (c) H_2 on Zeolite Na-Y at various temperatures computed using two different values for ρ_s. The filled symbols correspond to n^{ex} computed using the NIST reference value, $\rho_s = 2523\ \mathrm{kg\,m^{-3}}$. The empty symbols correspond to n^{ex} computed using the measured value in this study, $\rho_s = 2410\ \mathrm{kg\,m^{-3}}$. Panels d-f show the percentage relative deviation between the excess isotherms computed using the measured experimental value for ρ_s in this study relative to those calculated using the NIST reference value for ρ_s.

S6 Absolute isotherms and SSI model fits in units of $\mathrm{mol\,kg^{-1}}$

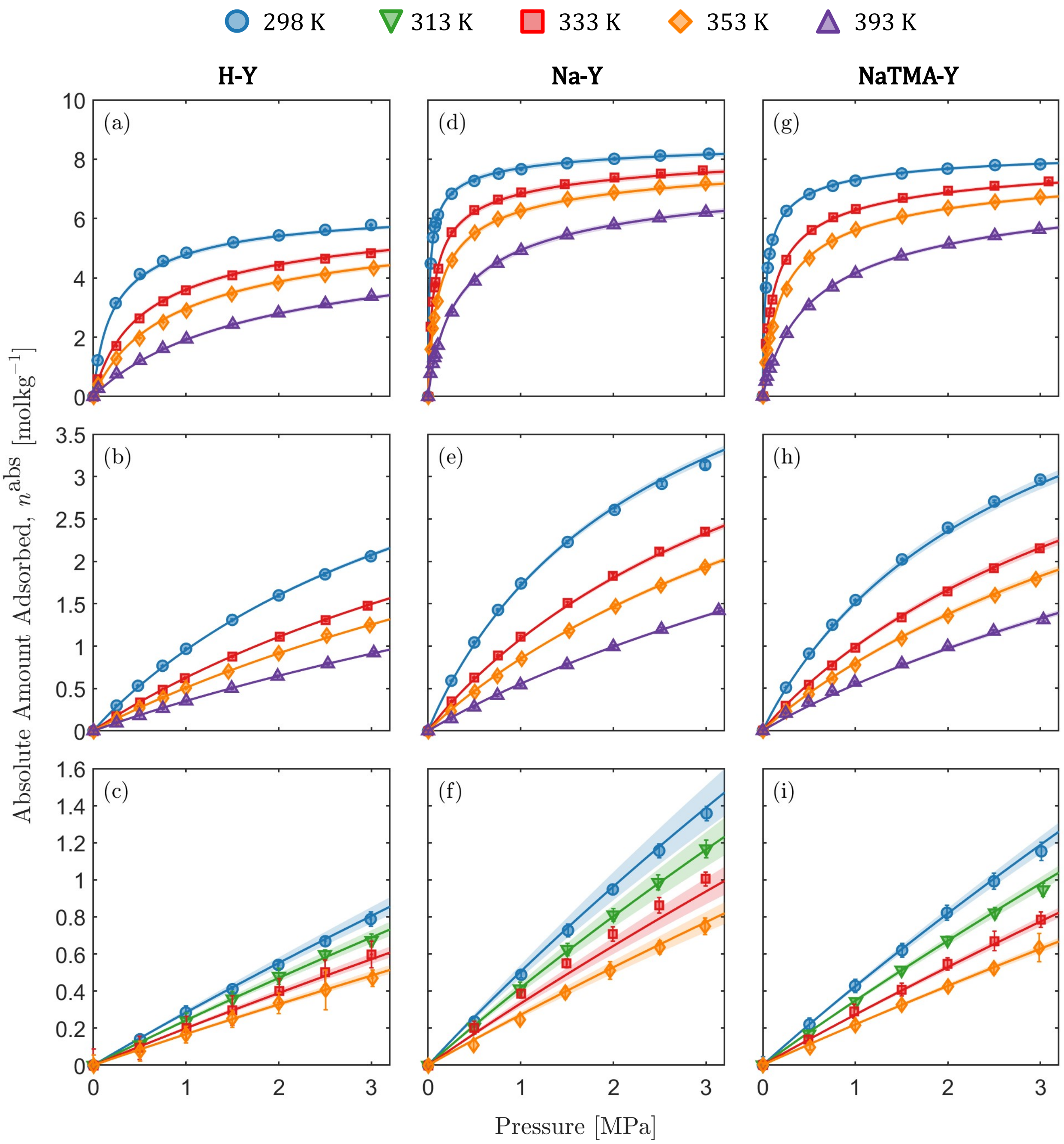

Figure S6: Absolute adsorption isotherms of (a, d, g) CO_2, (b, e, h) N_2, and (c, f, i) H_2 on Zeolite H-Y (a-c), Na-Y (d-f), and NaTMA-Y (g-i), at various temperatures. The solid lines represent the isotherm fitting to the simplified statistical isotherm (SSI) model (eq 10 of the main text), and the shaded regions show the 95% confidence bounds.

S7 Low pressure volumetric CO_2 adsorption isotherms

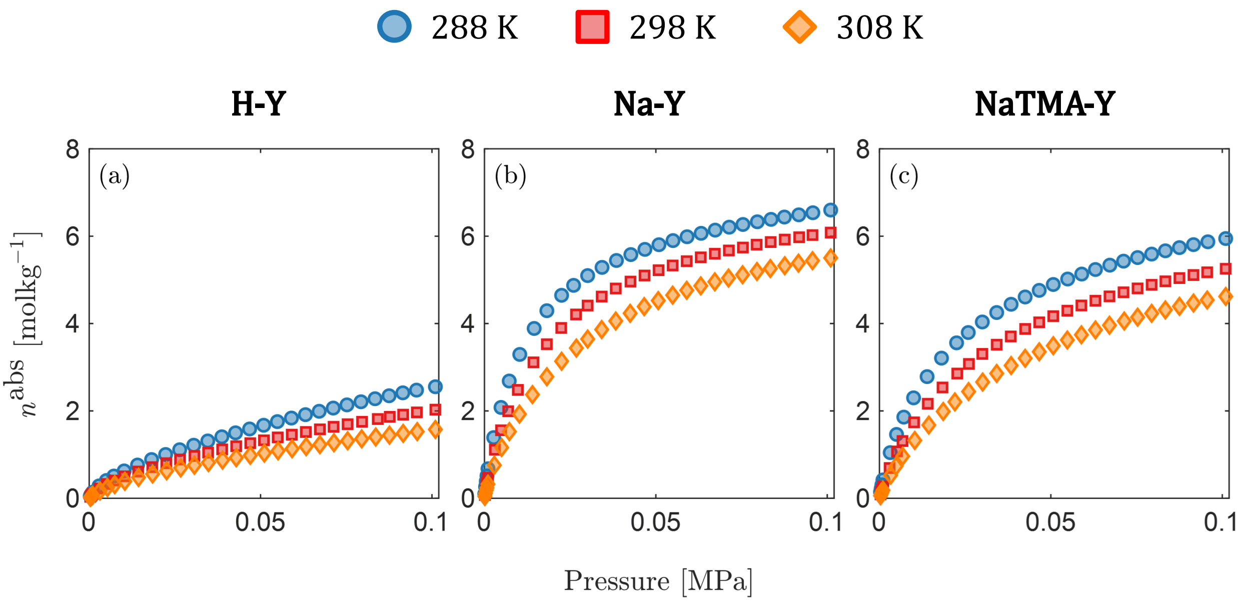

Figure S7: Excess adsorption isotherms of CO_2 on (a) Zeolite H-Y, (b) Na-Y, and (c) NaTMA-Y measured up to 0.1 MPa at 288 K, 298 K and 308 K using volumetry.

S8 Comparison of single-site Langmuir (SSL) and SSI models fits for absolute adsorption of N_2 and H_2

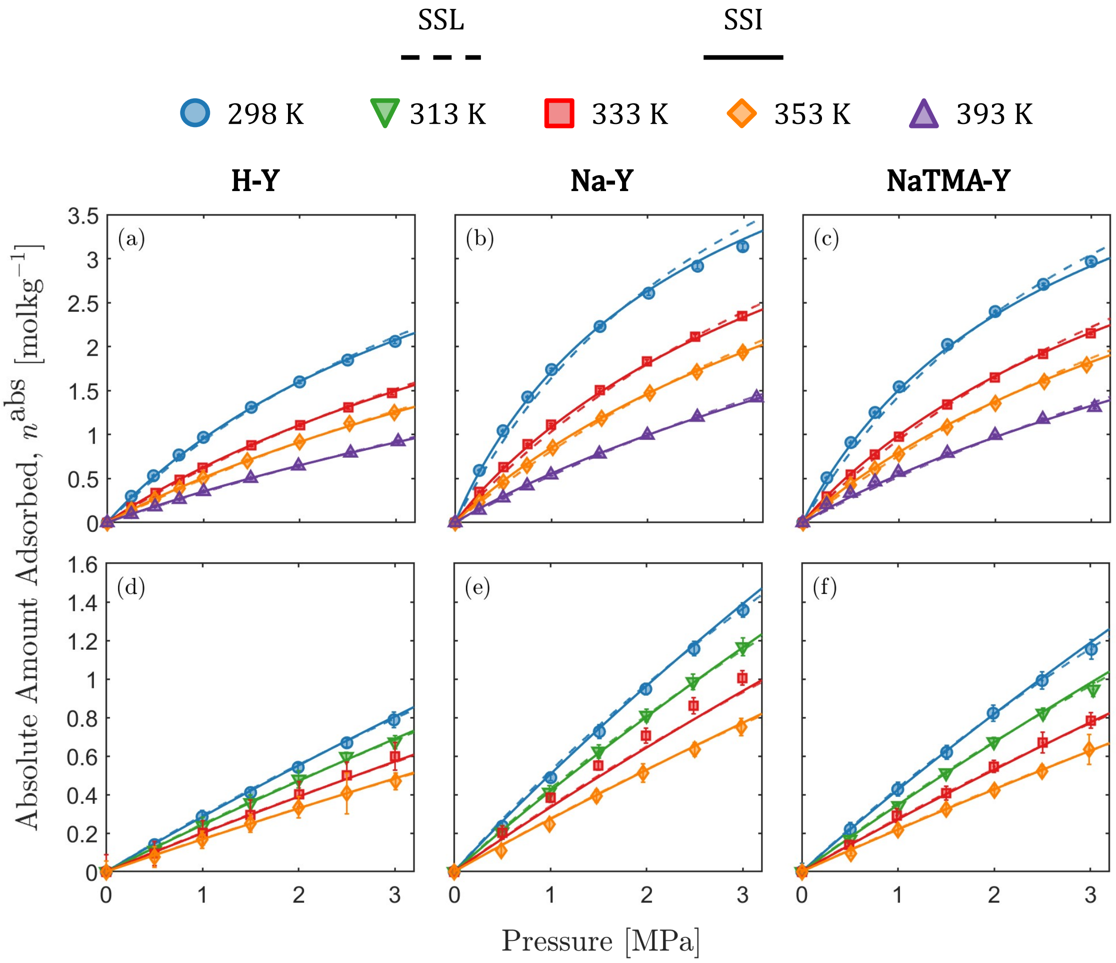

Figure S8: Absolute adsorption isotherms of N_2 and H_2 on Zeolite (a,d) H-Y, (b,e) Na-Y, and (c,f) NaTMA-Y, comparing the single-site Langmuir (dashed lines) and simplified statistical isotherm (solid lines) models.

S9 Dual-site Langmuir (DSL) model parameters for absolute adsorption of CO_2, N_2 and H_2

We compare the SSI model with the commonly used dual-site Langmuir (DSL) model[4]. The model is given by:

$$
\begin{aligned}
n^{\text{abs}} &= \frac{n_{\text{s,b}} b(T) p}{1 + b(T)p} + \frac{n_{\text{s,d}} d(T) p}{1 + d(T)p} \\
b(T) &= b_0 \exp\left[\frac{-\Delta U_{\text{b}}}{RT}\right] \\
d(T) &= d_0 \exp\left[\frac{-\Delta U_{\text{d}}}{RT}\right]
\end{aligned}
\tag{S1}
$$

where $n_{\text{s,b}}$ and $n_{\text{s,d}}$ [mol kg^{-1}] are the saturation capacities for the two sites (fixed for all sorbates for a given material and obtained by fitting the CO_2 isotherms first), b and d [bar^{-1}] are the temperature dependent adsorption coefficients, described by an Arrhenius expression with two constants, b_0 and d_0 [bar^{-1}] and $-\Delta U_{\text{b}}$ and $-\Delta U_{\text{d}}$ [kJ mol^{-1}] for the two sites respectively. The six fitted parameters for CO_2 are $n_{\text{s,b}}$, $n_{\text{s,d}}$, b_0, d_0, $-\Delta U_{\text{b}}$ and $-\Delta U_{\text{d}}$. For N_2 b_0, d_0, $-\Delta U_{\text{b}}$ and $-\Delta U_{\text{d}}$ were fitted, and for H_2 b and d were assumed to be equal as the isotherms are linear and b_0 and $-\Delta U_{\text{b}}$ were fitted.

Table S1: Dual-Site Langmuir (DSL) isotherm model parameters derived from fitting the CO_2, N_2 and H_2 isotherms. The values in parentheses represent the uncertainty values.

	**Dual-site Langmuir**					
	$n_{\text{s,b}}$ [mol kg^{-1}]	$b_0 \times 10^7$ [bar^{-1}]	$-\Delta U_{\text{b}}$ [kJ mol^{-1}]	$n_{\text{s,d}}$ [mol kg^{-1}]	$d_0 \times 10^7$ [bar^{-1}]	$-\Delta U_{\text{d}}$ [kJ mol^{-1}]
			**H-Y**			
CO_2	4.29 (0.05)	895.35 (32.75)	21.94 (0.11)	2.11 (0.11)	7.61 (0.74)	29.71 (0.27)
N_2		142.45 (16.94)	10.22 (0.03)		193.40 (19.65)	13.29 (0.03)
H_2		1311.45 (49.33)	8.90 (0.10)		1311.45 (100.47)	8.90 (0.21)
			**Na-Y**			
CO_2	4.61 (0.08)	34.14 (1.45)	32.94 (0.13)	314.21 (0.05)	314.21 (21.08)	33.12 (0.21)
N_2		807.97 (6.26)	16.32 (0.02)		8113.55 (399.87)	3.04 (0.15)
H_2		972.10 (60.32)	10.66 (0.17)		972.10 (88.44)	10.66 (0.25)
			**NaTMA-Y**			
CO_2	4.67 (0.05)	47.58 (1.38)	31.05 (0.16)	2.99 (0.03)	340.70 (17.38)	30.52 (0.21)
N_2		59.13 (2.86)	18.67 (0.13)		7416.79 (169.35)	18.67 (0.13)
H_2		694.64 (15.06)	11.04 (0.06)		694.64 (23.46)	11.04 (0.09)

S10 Comparison of dual-site Langmuir (DSL) and SSI models fits for absolute adsorption of CO_2, N_2 and H_2

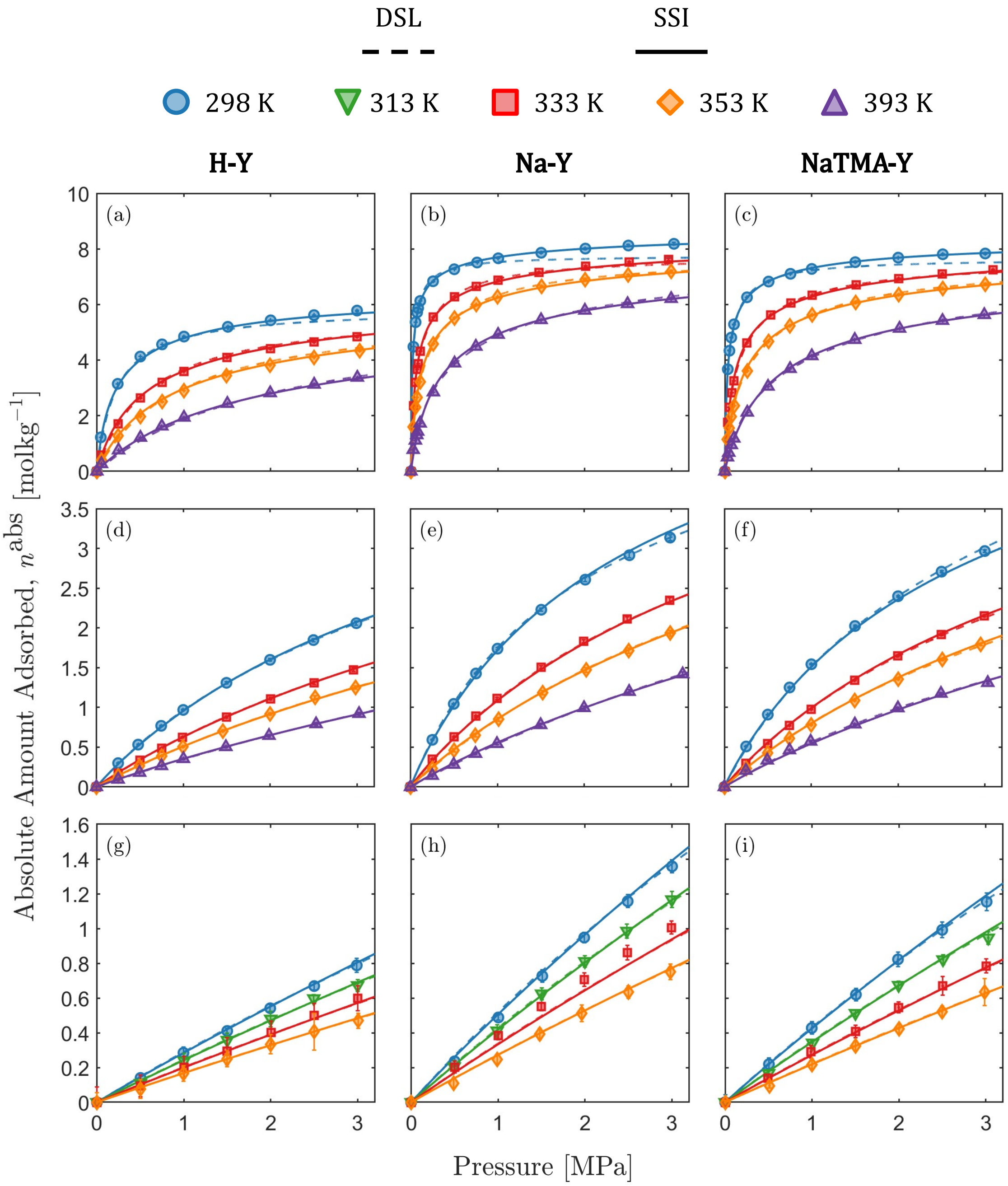

Figure S9: Absolute adsorption isotherms of CO_2, N_2 and H_2 on Zeolite (a,d,g) H-Y, (b,e,h) Na-Y, and (c,f,i) NaTMA-Y, comparing the dual-site Langmuir (dashed lines) and simplified statistical isotherm (solid lines) models.

S11 Relative deviation plots for SSL and SSI models

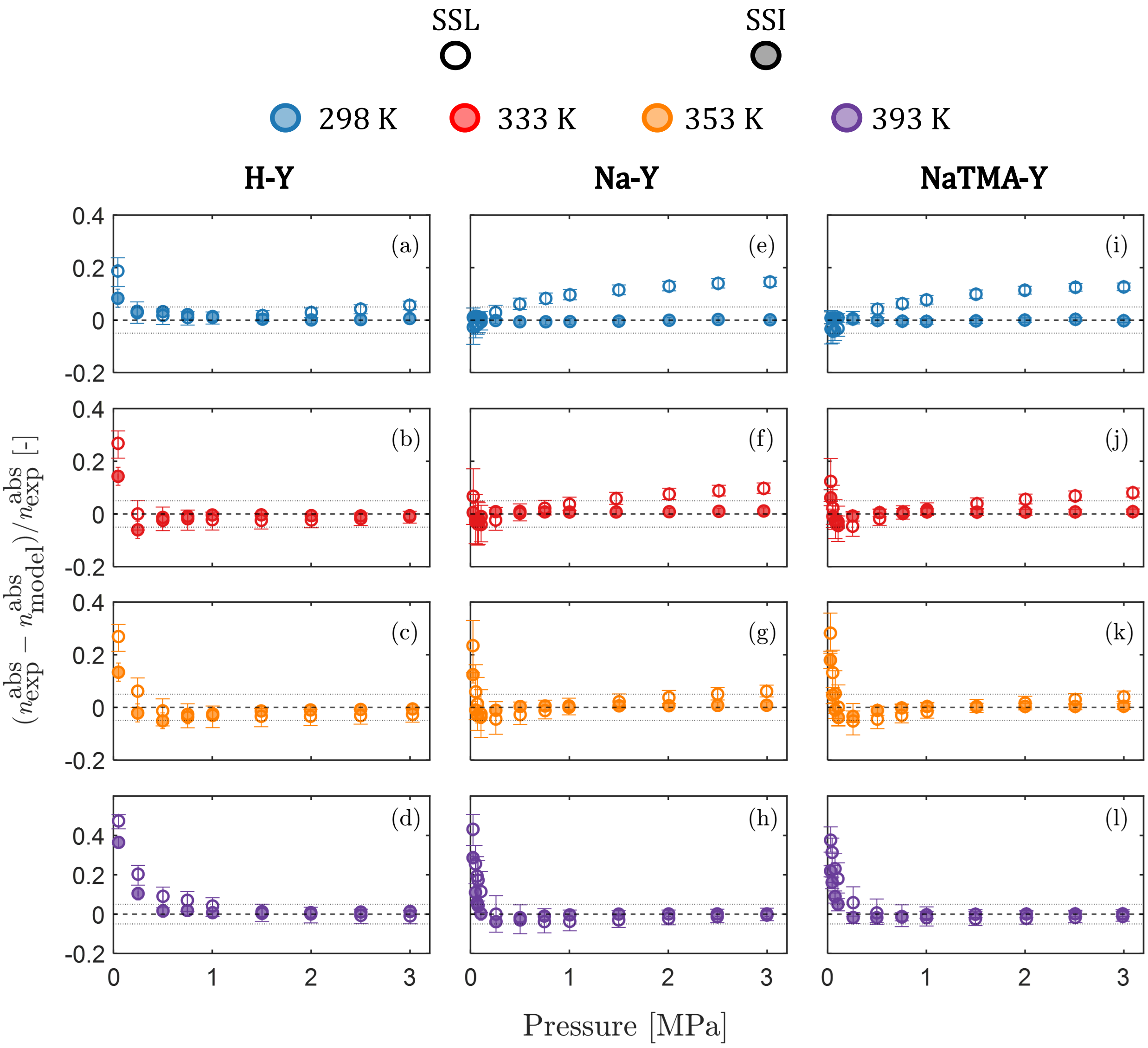

Figure S10: Relative deviation of the model fits (SSL: empty, and SSI: filled circles) for CO_2 on Zeolite (a-d) H-Y, (e-h) Na-Y, and (i-l) NaTMA-Y. The dashed lines correspond to 5% relative deviation.

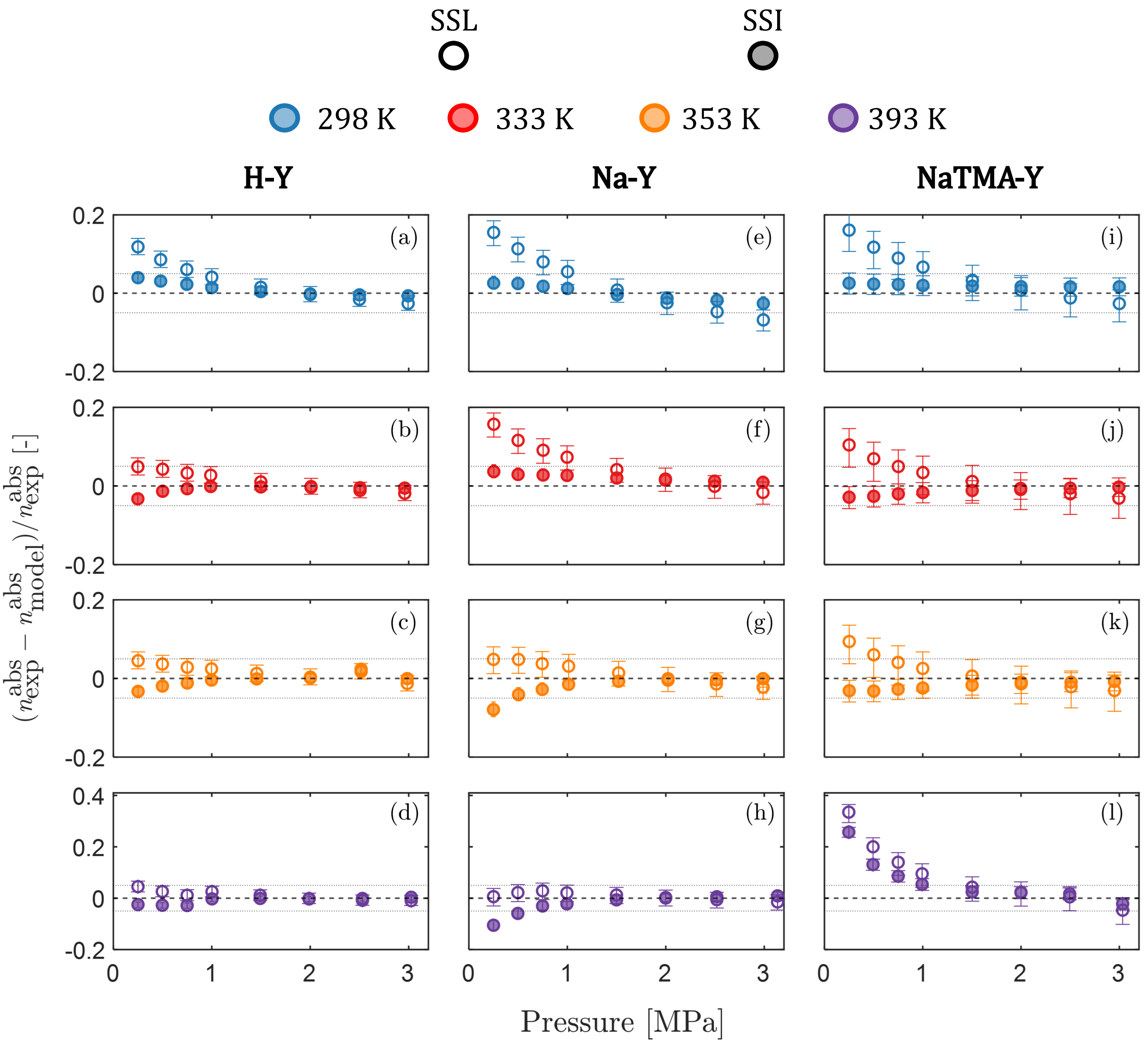

Figure S11: Relative deviation of the model fits (SSL: empty, and SSI: filled circles) for N_2 on Zeolite (a-d) H-Y, (e-h) Na-Y, and (i-l) NaTMA-Y. The dashed lines correspond to 5% relative deviation.

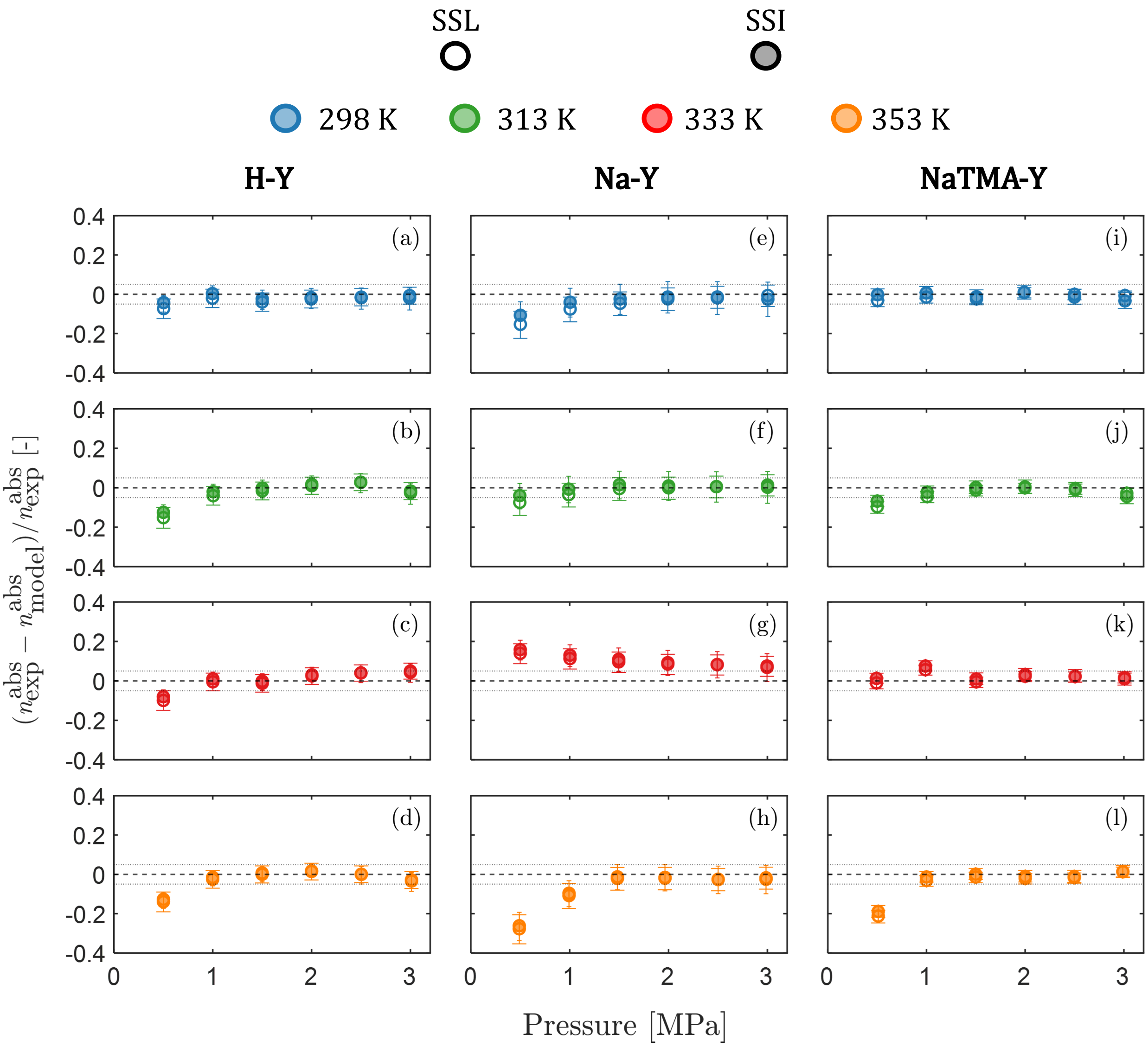

Figure S12: Relative deviation of the model fits (SSL: empty, and SSI: filled circles) for H_2 on Zeolite (a-d) H-Y, (e-h) Na-Y, and (i-l) NaTMA-Y. The dashed lines correspond to 5% relative deviation.

S12 Virial isotherm model and fitted parameters

The virial equation is given by the following equation[5,6] for each component i:

$$\ln(p/n_i) = \frac{1}{T}\sum_{j=0}^{M_1} a_j n_i^j + \sum_{j=0}^{M_2} b_j n_i^j \tag{S2}$$

where a and b are the virial coefficients for a gas-solid system. In this study, we used $M_1 = 3$ and $M_2 = 1$ to provide a good fit. The Henry constant K can be approximated from a_0 and b_0 using the following equation:

$$K = \exp\left(-\frac{a_0}{T} - b_0\right) \tag{S3}$$

The equilibrium data were fitted to the virial equation and the uncertainty bounds were calculated using the same procedure described in Section 2.4.2 of the main text. The fitted parameters for the three materials are listed below in Table S2.

Table S2: Virial isotherm model parameters derived from fitting the low pressure volumetric CO_2 equilibrium data shown in Figure S7. The values in parentheses represent the uncertainty values where available.

	Virial isotherm model					
	a_0 [K]	a_1 [$\mathrm{K\,kg\,mol^{-1}}$]	a_2 [$\mathrm{K\,kg^2\,mol^{-2}}$]	a_3 [$\mathrm{K\,kg^3\,mol^{-3}}$]	b_0 [−]	b_1 [$\mathrm{kg\,mol^{-1}}$]
**H-Y**	-3886.67 (13.34)	691.93 (12.51)	-317.84 (2.05)	57.77 (0.56)	10.59 (0.04)	-0.10 (0.04)
**Na-Y**	-3787.26 (13.32)	7.25 (3.27)	-30.43 (0.32)	3.89 (0.03)	8.64 (0.04)	0.49 (0.04)
**NaTMA-Y**	-3889.31 (7.09)	20.02 (2.13)	-6.72 (0.20)	1.33 (0.02)	9.68 (0.02)	0.25 (0.07)

S13 Excess, net, and absolute isotherm data for H-Y, Na-Y and NaTMA-Y

Table S3: Excess, net, and absolute adsorbed amounts of CO_2, N_2, and H_2 on Zeolite H-Y, Na-Y, and NaTMA-Y at different temperatures and the uncertainty values associated with them denoted by δ.

H-Y

Gas	Temperature [K]	Pressure [MPa]	Density [kg/m^3]	n^{ex}	n^{net}	n^{abs} [mol/kg]	δn^{ex}	δn^{net}	δn^{abs}
CO_2	298.15	0.000	0.000	0.000	0.000	0.000	0.002	0.002	0.002
		0.046	0.818	1.217	1.209	1.222	0.001	0.001	0.001
		0.240	4.322	3.122	3.076	3.147	0.002	0.001	0.002
		0.501	9.112	4.075	3.978	4.129	0.004	0.002	0.004
		0.751	13.845	4.484	4.336	4.566	0.004	0.001	0.004
		1.004	18.759	4.737	4.537	4.847	0.006	0.001	0.006
		1.509	29.030	5.026	4.717	5.198	0.009	0.002	0.009
		2.002	39.714	5.197	4.774	5.432	0.012	0.002	0.012
		2.501	51.323	5.316	4.769	5.619	0.015	0.002	0.015
		2.997	63.787	5.405	4.725	5.782	0.019	0.002	0.019
	333.15	0.000	0.000	0.000	0.000	0.000	0.002	0.002	0.002
		0.048	0.766	0.585	0.577	0.590	0.001	0.001	0.001
		0.247	3.942	1.688	1.646	1.711	0.004	0.003	0.004
		0.500	8.033	2.585	2.500	2.633	0.003	0.001	0.003
		0.752	12.195	3.133	3.003	3.205	0.004	0.001	0.004
		1.000	16.368	3.492	3.317	3.588	0.005	0.002	0.005
		1.496	24.931	3.940	3.674	4.087	0.007	0.002	0.007
		2.002	34.033	4.210	3.847	4.411	0.010	0.002	0.010
		2.502	43.399	4.391	3.929	4.648	0.013	0.002	0.013
		2.997	53.093	4.522	3.956	4.836	0.016	0.002	0.016
	353.15	0.000	0.000	0.000	0.000	0.000	0.002	0.002	0.002
		0.052	0.776	0.403	0.395	0.408	0.004	0.004	0.004
		0.246	3.674	1.248	1.209	1.270	0.002	0.002	0.002
		0.499	7.509	1.929	1.849	1.973	0.004	0.003	0.004
		0.751	11.401	2.435	2.313	2.502	0.005	0.003	0.005
		1.004	15.352	2.812	2.649	2.903	0.005	0.002	0.005
		1.495	23.195	3.311	3.063	3.448	0.008	0.004	0.008
		1.994	31.411	3.637	3.302	3.822	0.010	0.003	0.010
		2.500	40.045	3.866	3.439	4.102	0.012	0.003	0.012
		3.025	49.304	4.040	3.514	4.331	0.015	0.002	0.015
	393.15	0.000	0.000	0.000	0.000	0.000	0.001	0.001	0.001
		0.053	0.707	0.262	0.254	0.266	0.001	0.001	0.001
		0.250	3.335	0.742	0.707	0.762	0.002	0.002	0.002
		0.502	6.738	1.175	1.103	1.215	0.004	0.003	0.004

		0.748	10.101	1.562	1.454	1.621	0.004	0.002	0.004
		1.003	13.628	1.858	1.713	1.938	0.004	0.002	0.004
		1.503	20.607	2.313	2.094	2.435	0.006	0.002	0.007
		1.997	27.670	2.654	2.359	2.817	0.008	0.002	0.008
		2.503	35.049	2.919	2.545	3.126	0.011	0.003	0.011
		3.000	42.476	3.125	2.672	3.376	0.014	0.006	0.014
N_2	298.15	0.000	0.000	0.000	0.000	0.000	0.001	0.001	0.001
		0.250	2.835	0.273	0.225	0.299	0.002	0.001	0.003
		0.481	5.439	0.482	0.391	0.532	0.004	0.001	0.004
		0.746	8.437	0.690	0.549	0.769	0.006	0.001	0.006
		1.000	11.302	0.864	0.674	0.968	0.008	0.001	0.008
		1.498	16.940	1.152	0.868	1.309	0.012	0.003	0.012
		2.000	22.624	1.388	1.010	1.598	0.016	0.001	0.016
		2.500	28.288	1.585	1.111	1.847	0.020	0.002	0.020
		2.994	33.878	1.745	1.177	2.059	0.024	0.002	0.024
	333.15	0.000	0.000	0.000	0.000	0.000	0.004	0.003	0.004
		0.249	2.507	0.150	0.108	0.174	0.007	0.007	0.007
		0.501	5.034	0.290	0.206	0.337	0.005	0.003	0.005
		0.750	7.534	0.417	0.290	0.487	0.008	0.005	0.008
		0.989	9.937	0.530	0.363	0.622	0.008	0.004	0.008
		1.498	15.047	0.740	0.488	0.880	0.011	0.003	0.011
		2.008	20.161	0.922	0.584	1.109	0.015	0.004	0.015
		2.506	25.146	1.076	0.655	1.309	0.018	0.003	0.018
		2.960	29.684	1.199	0.702	1.475	0.021	0.003	0.021
	353.15	0.000	0.000	0.000	0.000	0.000	0.003	0.003	0.003
		0.251	2.388	0.117	0.077	0.139	0.003	0.003	0.003
		0.498	4.722	0.224	0.145	0.268	0.005	0.004	0.005
		0.751	7.104	0.325	0.206	0.391	0.007	0.004	0.007
		0.997	9.433	0.419	0.261	0.506	0.009	0.006	0.009
		1.456	13.759	0.576	0.345	0.703	0.010	0.003	0.010
		2.001	18.904	0.742	0.425	0.917	0.014	0.003	0.014
		2.517	23.870	0.903	0.503	1.125	0.018	0.007	0.018
		2.985	28.126	0.989	0.518	1.250	0.021	0.007	0.021
	393.15	0.000	0.000	0.000	0.000	0.000	0.004	0.004	0.004
		0.250	2.134	0.074	0.039	0.094	0.004	0.003	0.004
		0.497	4.214	0.142	0.071	0.181	0.005	0.003	0.005
		0.750	6.332	0.206	0.100	0.264	0.007	0.006	0.008
		1.002	8.478	0.275	0.133	0.353	0.008	0.005	0.008
		1.491	12.585	0.387	0.176	0.504	0.011	0.005	0.011
		1.988	16.755	0.490	0.209	0.645	0.012	0.003	0.012
		2.530	21.286	0.594	0.238	0.792	0.015	0.003	0.015
		3.025	25.427	0.685	0.259	0.921	0.018	0.004	0.018

H_2	298.15	0.000	0.000	0.000	0.000	0.000	0.010	0.010	0.010
		0.503	0.408	0.087	-0.007	0.140	0.010	0.009	0.010
		0.997	0.806	0.181	-0.007	0.285	0.034	0.033	0.034
		1.501	1.210	0.254	-0.027	0.410	0.015	0.009	0.015
		1.996	1.604	0.334	-0.039	0.541	0.021	0.014	0.021
		2.502	2.005	0.410	-0.057	0.669	0.024	0.013	0.024
		2.992	2.391	0.480	-0.077	0.788	0.040	0.032	0.040
	313.15	0.000	0.000	0.000	0.000	0.016	0.016	0.016	0.016
		0.500	0.386	0.060	-0.030	0.024	0.024	0.023	0.024
		1.004	0.773	0.139	-0.041	0.043	0.043	0.042	0.043
		1.501	1.153	0.209	-0.059	0.040	0.040	0.039	0.040
		2.002	1.532	0.282	-0.075	0.044	0.044	0.041	0.044
		2.497	1.906	0.351	-0.093	0.024	0.024	0.015	0.024
		3.001	2.285	0.378	-0.154	0.035	0.035	0.026	0.035
	333.15	0.000	0.000	0.000	0.000	0.000	0.087	0.087	0.087
		0.500	0.363	0.047	-0.038	0.094	0.062	0.062	0.062
		1.001	0.725	0.108	-0.060	0.202	0.063	0.062	0.063
		1.501	1.083	0.155	-0.097	0.295	0.078	0.077	0.078
		2.003	1.442	0.216	-0.120	0.402	0.065	0.064	0.065
		2.499	1.794	0.270	-0.148	0.501	0.067	0.065	0.067
		3.000	2.148	0.321	-0.179	0.598	0.071	0.068	0.071
	353.15	0.000	0.000	0.000	0.000	0.054	0.054	0.054	0.054
		0.501	0.343	0.031	-0.048	0.053	0.053	0.053	0.053
		0.999	0.682	0.077	-0.082	0.044	0.044	0.044	0.044
		1.500	1.022	0.119	-0.119	0.045	0.045	0.044	0.045
		1.999	1.358	0.159	-0.157	0.054	0.054	0.053	0.054
		2.503	1.696	0.188	-0.206	0.106	0.106	0.105	0.106
		3.011	2.035	0.207	-0.267	0.044	0.044	0.039	0.044

Na-Y

Gas	Temperature [K]	Pressure [MPa]	Density [kg/m^3]	n^{ex}	n^{net}	n^{abs} [mol/kg]	δn^{ex}	δn^{net}	δn^{abs}
CO_2	298.15	0.000	0.000	0.000	0.000	0.000	0.003	0.003	0.004
		0.028	0.389	4.477	4.474	4.481	0.002	0.002	0.003
		0.053	0.827	5.358	5.350	5.364	0.002	0.002	0.003
		0.072	1.156	5.709	5.698	5.719	0.003	0.002	0.003
		0.083	1.349	5.860	5.847	5.871	0.003	0.002	0.003
		0.107	1.788	6.115	6.098	6.129	0.003	0.002	0.003
		0.254	4.432	6.800	6.759	6.836	0.008	0.007	0.008
		0.499	8.917	7.196	7.112	7.268	0.007	0.004	0.007
		0.762	13.870	7.399	7.268	7.512	0.010	0.002	0.010
		1.006	18.603	7.511	7.336	7.663	0.013	0.002	0.013

	1.500	28.607	7.628	7.359	7.862	0.020	0.003	0.020
	2.009	39.619	7.689	7.316	8.012	0.028	0.002	0.028
	2.504	51.082	7.706	7.225	8.123	0.035	0.003	0.036
	3.029	64.244	7.659	7.054	8.183	0.045	0.003	0.045
333.15	0.000	0.000	0.000	0.000	0.000	0.001	0.000	0.001
	0.028	0.447	2.347	2.343	2.351	0.001	0.001	0.001
	0.053	0.835	3.188	3.180	3.195	0.001	0.001	0.001
	0.072	1.147	3.663	3.652	3.673	0.002	0.001	0.002
	0.081	1.286	3.854	3.842	3.864	0.001	0.001	0.002
	0.107	1.705	4.299	4.283	4.313	0.002	0.002	0.002
	0.255	4.041	5.509	5.471	5.542	0.004	0.003	0.004
	0.500	8.006	6.216	6.140	6.281	0.006	0.001	0.006
	0.751	12.138	6.546	6.431	6.645	0.009	0.002	0.009
	1.001	16.322	6.744	6.591	6.877	0.011	0.001	0.011
	1.473	24.447	6.964	6.734	7.164	0.017	0.001	0.017
	2.009	34.054	7.097	6.776	7.375	0.024	0.003	0.024
	2.512	43.443	7.168	6.758	7.522	0.030	0.002	0.030
	2.965	52.264	7.200	6.708	7.626	0.036	0.002	0.036
353.15	0.000	0.000	0.000	0.000	0.000	0.007	0.007	0.008
	0.025	0.383	1.587	1.583	1.590	0.005	0.005	0.006
	0.053	0.791	2.289	2.282	2.295	0.006	0.006	0.006
	0.071	1.064	2.649	2.639	2.658	0.006	0.006	0.006
	0.106	1.581	3.204	3.189	3.217	0.006	0.006	0.006
	0.257	3.824	4.553	4.517	4.584	0.007	0.006	0.007
	0.503	7.538	5.460	5.389	5.521	0.008	0.006	0.008
	0.754	11.396	5.896	5.789	5.989	0.010	0.005	0.010
	0.995	15.154	6.149	6.006	6.272	0.012	0.005	0.012
	1.503	23.241	6.461	6.242	6.651	0.017	0.005	0.017
	2.006	31.520	6.633	6.336	6.890	0.023	0.006	0.023
	2.499	39.882	6.736	6.360	7.061	0.028	0.005	0.028
	2.994	48.552	6.798	6.341	7.194	0.034	0.007	0.034
393.15	0.000	0.000	0.000	0.000	0.000	0.006	0.005	0.006
	0.025	0.354	0.774	0.771	0.777	0.005	0.004	0.005
	0.051	0.677	1.106	1.100	1.112	0.004	0.004	0.004
	0.067	0.897	1.300	1.292	1.308	0.004	0.004	0.004
	0.077	1.035	1.424	1.415	1.433	0.005	0.005	0.005
	0.105	1.406	1.715	1.701	1.726	0.004	0.004	0.005
	0.258	3.459	2.819	2.786	2.847	0.005	0.004	0.005
	0.502	6.721	3.834	3.770	3.889	0.007	0.005	0.007
	0.747	10.057	4.409	4.314	4.491	0.008	0.004	0.008
	1.004	13.594	4.809	4.681	4.920	0.010	0.004	0.010
	1.498	20.506	5.280	5.087	5.447	0.015	0.004	0.015
	2.003	27.674	5.561	5.300	5.786	0.020	0.004	0.020

		2.495	34.814	5.739	5.411	6.023	0.024	0.004	0.024
		2.997	42.267	5.864	5.466	6.208	0.030	0.004	0.030
N_2	298.15	0.000	0.000	0.000	0.000	0.000	0.001	0.001	0.001
		0.252	2.842	0.556	0.514	0.592	0.003	0.001	0.003
		0.498	5.619	0.972	0.889	1.044	0.006	0.001	0.006
		0.754	8.508	1.318	1.192	1.427	0.009	0.002	0.009
		1.002	11.313	1.594	1.426	1.739	0.012	0.002	0.012
		1.506	17.038	2.010	1.758	2.229	0.019	0.004	0.019
		2.012	22.760	2.315	1.979	2.607	0.025	0.003	0.025
		2.521	28.527	2.549	2.127	2.914	0.031	0.002	0.031
		2.990	33.826	2.703	2.202	3.136	0.037	0.004	0.037
	333.15	0.000	0.000	0.000	0.000	0.000	0.005	0.005	0.005
		0.253	2.534	0.316	0.278	0.348	0.005	0.004	0.005
		0.500	5.022	0.566	0.492	0.630	0.007	0.003	0.007
		0.754	7.575	0.792	0.680	0.889	0.009	0.004	0.009
		0.997	10.025	0.984	0.835	1.112	0.011	0.003	0.012
		1.502	15.090	1.314	1.091	1.508	0.017	0.004	0.017
		1.996	20.042	1.574	1.278	1.831	0.022	0.004	0.022
		2.496	25.051	1.791	1.420	2.112	0.028	0.004	0.028
		2.987	29.962	1.966	1.522	2.350	0.033	0.004	0.033
	353.15	0.000	0.000	0.000	0.000	0.000	0.025	0.024	0.026
		0.249	2.359	0.203	0.168	0.234	0.018	0.017	0.019
		0.504	4.775	0.396	0.326	0.457	0.019	0.017	0.019
		0.745	7.048	0.559	0.455	0.649	0.019	0.017	0.020
		1.013	9.580	0.727	0.585	0.850	0.021	0.017	0.021
		1.521	14.375	0.999	0.787	1.184	0.024	0.017	0.024
		2.022	19.099	1.226	0.943	1.470	0.027	0.017	0.028
		2.514	23.723	1.412	1.061	1.716	0.031	0.017	0.032
		2.988	28.305	1.571	1.152	1.933	0.036	0.017	0.036
	393.15	0.000	0.000	0.000	0.000	0.000	0.012	0.012	0.013
		0.250	2.121	0.115	0.084	0.142	0.009	0.009	0.010
		0.497	4.206	0.228	0.166	0.282	0.011	0.009	0.011
		0.748	6.350	0.338	0.244	0.419	0.011	0.009	0.012
		0.996	8.431	0.435	0.310	0.543	0.013	0.009	0.013
		1.499	12.673	0.618	0.431	0.780	0.016	0.009	0.017
		1.999	16.873	0.778	0.528	0.994	0.020	0.009	0.021
		2.518	21.234	0.926	0.612	1.198	0.025	0.009	0.025
		3.135	26.385	1.081	0.691	1.419	0.030	0.009	0.030
H_2	298.15	0.000	0.000	0.000	0.000	0.000	0.012	0.012	0.012
		0.501	0.406	0.163	0.080	0.236	0.025	0.025	0.025
		1.004	0.812	0.343	0.176	0.488	0.016	0.011	0.016
		1.510	1.217	0.510	0.260	0.727	0.035	0.030	0.035

		1.993	1.602	0.663	0.333	0.948	0.029	0.016	0.029
		2.493	1.998	0.801	0.390	1.157	0.037	0.022	0.037
		3.004	2.401	0.930	0.437	1.358	0.038	0.013	0.038
	313.15	0.000	0.000	0.000	0.000	0.000	0.033	0.033	0.033
		0.495	0.382	0.137	0.058	0.205	0.035	0.035	0.035
		0.990	0.762	0.276	0.119	0.412	0.036	0.034	0.036
		1.502	1.153	0.419	0.182	0.624	0.034	0.029	0.034
		2.000	1.531	0.538	0.223	0.811	0.033	0.024	0.033
		2.484	1.896	0.648	0.258	0.985	0.040	0.028	0.040
		3.001	2.285	0.760	0.290	1.166	0.046	0.031	0.046
	333.15	0.000	0.000	0.000	0.000	0.000	0.019	0.019	0.019
		0.500	0.363	0.138	0.063	0.202	0.034	0.033	0.034
		1.003	0.726	0.255	0.106	0.384	0.024	0.021	0.024
		1.496	1.080	0.358	0.136	0.550	0.024	0.017	0.024
		1.993	1.435	0.451	0.156	0.707	0.038	0.032	0.038
		2.492	1.789	0.543	0.175	0.862	0.042	0.032	0.042
		2.996	2.145	0.623	0.182	1.005	0.037	0.018	0.037
	353.15	0.000	0.000	0.000	0.000	0.000	0.032	0.032	0.032
		0.490	0.335	0.048	-0.021	0.108	0.027	0.026	0.027
		0.993	0.678	0.126	-0.013	0.247	0.026	0.024	0.026
		1.482	1.010	0.213	0.005	0.393	0.033	0.029	0.033
		1.964	1.335	0.274	-0.001	0.511	0.047	0.043	0.047
		2.502	1.695	0.333	-0.015	0.635	0.036	0.025	0.036
		2.986	2.018	0.390	-0.025	0.750	0.045	0.033	0.045

NaTMA-Y

Gas	Temperature [K]	Pressure [MPa]	Density [kg/m^3]	n^{ex}	n^{net}	n^{abs} [mol/kg]	δn^{ex}	δn^{net}	δn^{abs}
CO_2	298.15	0.000	0.000	0.000	0.000	0.000	0.011	0.010	0.011
		0.034	0.590	3.667	3.661	3.672	0.008	0.007	0.008
		0.053	0.924	4.331	4.322	4.339	0.008	0.008	0.009
		0.074	1.296	4.801	4.789	4.811	0.008	0.007	0.008
		0.106	1.857	5.273	5.255	5.287	0.008	0.007	0.008
		0.256	4.555	6.223	6.179	6.259	0.008	0.007	0.008
		0.504	9.130	6.754	6.664	6.826	0.008	0.007	0.008
		0.757	13.900	6.996	6.859	7.104	0.009	0.007	0.009
		1.001	18.638	7.134	6.951	7.280	0.009	0.007	0.010
		1.503	28.799	7.298	7.014	7.523	0.011	0.007	0.012
		1.997	39.494	7.377	6.988	7.686	0.014	0.007	0.014
		2.511	51.366	7.405	6.899	7.807	0.017	0.007	0.017
		2.999	63.631	7.337	6.710	7.835	0.020	0.007	0.020
	333.15	0.000	0.000	0.000	0.000	0.000	0.002	0.002	0.002

		0.032	0.513	1.761	1.756	1.765	0.002	0.002	0.002
		0.053	0.846	2.295	2.287	2.302	0.002	0.002	0.002
		0.079	1.260	2.818	2.806	2.828	0.003	0.003	0.003
		0.106	1.682	3.245	3.228	3.258	0.003	0.003	0.003
		0.253	4.030	4.580	4.540	4.611	0.002	0.002	0.002
		0.527	8.453	5.555	5.471	5.621	0.003	0.002	0.004
		0.764	12.363	5.956	5.834	6.052	0.004	0.002	0.004
		1.006	16.440	6.205	6.043	6.334	0.005	0.002	0.005
		1.513	25.184	6.506	6.257	6.702	0.007	0.002	0.008
		2.004	34.018	6.667	6.332	6.933	0.010	0.002	0.010
		2.507	43.437	6.764	6.336	7.104	0.013	0.002	0.013
		3.091	54.901	6.827	6.286	7.256	0.016	0.002	0.016
	353.15	0.000	0.000	0.000	0.000	0.000	0.002	0.002	0.002
		0.028	0.420	1.136	1.132	1.139	0.003	0.003	0.003
		0.051	0.766	1.559	1.552	1.565	0.003	0.003	0.004
		0.078	1.153	1.957	1.946	1.966	0.006	0.006	0.006
		0.108	1.616	2.341	2.326	2.354	0.002	0.002	0.003
		0.257	3.836	3.587	3.549	3.617	0.002	0.002	0.002
		0.504	7.572	4.608	4.533	4.667	0.003	0.002	0.003
		0.750	11.362	5.151	5.039	5.239	0.004	0.002	0.004
		1.008	15.384	5.507	5.355	5.627	0.009	0.008	0.009
		1.509	23.387	5.892	5.661	6.074	0.008	0.003	0.008
		2.000	31.493	6.111	5.801	6.358	0.009	0.002	0.009
		2.508	40.115	6.254	5.858	6.567	0.012	0.002	0.012
		2.999	48.760	6.345	5.865	6.726	0.014	0.002	0.014
	393.15	0.000	0.000	0.000	0.000	0.000	0.005	0.005	0.005
		0.030	0.590	0.503	0.499	0.506	0.004	0.003	0.004
		0.046	0.924	0.670	0.664	0.674	0.004	0.004	0.005
		0.075	1.296	0.941	0.931	0.949	0.004	0.003	0.004
		0.104	1.857	1.174	1.160	1.184	0.004	0.004	0.004
		0.259	4.555	2.094	2.060	2.121	0.004	0.003	0.004
		0.498	9.130	3.000	2.934	3.052	0.004	0.004	0.004
		0.752	13.900	3.610	3.510	3.689	0.005	0.004	0.005
		1.002	18.638	4.044	3.911	4.150	0.011	0.010	0.012
		1.497	28.799	4.576	4.374	4.736	0.008	0.005	0.008
		2.013	39.494	4.921	4.647	5.139	0.009	0.004	0.009
		2.508	51.366	5.145	4.800	5.419	0.011	0.004	0.011
		2.989	63.631	5.300	4.884	5.630	0.013	0.005	0.013
N_2	298.15	0.000	0.000	0.000	0.000	0.000	0.001	0.001	0.001
		0.251	2.830	0.474	0.426	0.509	0.002	0.001	0.002
		0.500	5.648	0.841	0.745	0.910	0.003	0.002	0.004
		0.751	8.478	1.147	1.003	1.251	0.004	0.001	0.004
		1.002	11.318	1.404	1.212	1.543	0.005	0.001	0.005

		1.505	17.002	1.815	1.526	2.024	0.009	0.003	0.009
		2.001	22.626	2.121	1.736	2.398	0.011	0.003	0.011
		2.502	28.305	2.360	1.879	2.707	0.013	0.002	0.013
		3.000	33.949	2.550	1.973	2.967	0.016	0.002	0.016
	333.15	0.000	0.000	0.000	0.000	0.000	0.003	0.003	0.003
		0.251	2.531	0.263	0.220	0.294	0.003	0.002	0.003
		0.501	5.048	0.483	0.397	0.545	0.004	0.003	0.004
		0.752	7.557	0.679	0.551	0.772	0.005	0.003	0.005
		1.001	10.062	0.854	0.683	0.977	0.005	0.002	0.005
		1.503	15.106	1.156	0.900	1.342	0.008	0.003	0.008
		1.996	20.052	1.401	1.060	1.647	0.013	0.008	0.013
		2.501	25.088	1.611	1.185	1.919	0.016	0.010	0.016
		2.995	30.043	1.784	1.273	2.153	0.014	0.003	0.014
	353.15	0.000	0.000	0.000	0.000	0.000	0.011	0.010	0.011
		0.252	2.429	0.201	0.160	0.231	0.008	0.008	0.008
		0.501	4.787	0.370	0.289	0.429	0.010	0.009	0.010
		0.749	7.131	0.523	0.402	0.611	0.009	0.008	0.009
		1.000	9.497	0.664	0.502	0.780	0.009	0.008	0.009
		1.502	14.237	0.914	0.672	1.089	0.010	0.008	0.011
		2.002	18.932	1.126	0.804	1.358	0.012	0.008	0.012
		2.509	23.705	1.311	0.908	1.602	0.014	0.008	0.014
		2.950	27.837	1.450	0.977	1.792	0.015	0.007	0.015
	393.15	0.000	0.000	0.000	0.000	0.000	0.006	0.006	0.006
		0.248	2.085	0.180	0.145	0.206	0.005	0.005	0.005
		0.494	4.166	0.284	0.213	0.335	0.006	0.006	0.007
		0.750	6.343	0.386	0.278	0.463	0.006	0.004	0.006
		0.994	8.372	0.470	0.328	0.573	0.008	0.007	0.009
		1.503	12.609	0.634	0.420	0.789	0.008	0.004	0.008
		1.999	16.766	0.786	0.501	0.992	0.009	0.004	0.009
		2.495	20.912	0.918	0.562	1.175	0.011	0.005	0.011
		3.034	25.412	0.998	0.566	1.310	0.015	0.008	0.015
H_2	298.15	0.000	0.000	0.000	0.000	0.000	0.044	0.044	0.044
		0.504	0.409	0.148	0.064	0.218	0.035	0.035	0.035
		0.998	0.807	0.286	0.120	0.423	0.036	0.033	0.036
		1.506	1.214	0.417	0.167	0.624	0.036	0.032	0.036
		1.990	1.600	0.538	0.209	0.811	0.041	0.034	0.041
		2.499	2.003	0.654	0.242	0.995	0.044	0.032	0.044
		3.011	2.406	0.750	0.255	1.160	0.050	0.035	0.050
	313.15	0.000	0.000	0.000	0.000	0.000	0.004	0.004	0.004
		0.501	0.387	0.101	0.021	0.167	0.020	0.020	0.020
		1.007	0.775	0.215	0.056	0.347	0.017	0.012	0.017
		1.500	1.152	0.316	0.079	0.512	0.019	0.007	0.019
		1.998	1.529	0.410	0.096	0.671	0.023	0.004	0.023

	2.508	1.915	0.501	0.108	0.828	0.029	0.004	0.029
	3.030	2.306	0.577	0.103	0.971	0.036	0.010	0.036
333.15	0.000	0.000	0.000	0.000	0.000	0.030	0.030	0.030
	0.493	0.358	0.072	-0.002	0.133	0.023	0.022	0.023
	0.989	0.716	0.160	0.013	0.282	0.036	0.034	0.036
	1.504	1.086	0.225	0.002	0.410	0.035	0.031	0.035
	1.999	1.439	0.300	0.004	0.545	0.032	0.023	0.032
	2.506	1.799	0.368	-0.002	0.674	0.053	0.046	0.053
	3.007	2.153	0.423	-0.020	0.790	0.041	0.025	0.041
353.15	0.000	0.000	0.000	0.000	0.000	0.011	0.011	0.011
	0.511	0.350	0.044	-0.028	0.104	0.014	0.013	0.014
	1.000	0.683	0.099	-0.041	0.216	0.024	0.021	0.024
	1.499	1.021	0.149	-0.061	0.323	0.028	0.024	0.028
	2.000	1.359	0.191	-0.088	0.423	0.029	0.020	0.029
	2.497	1.692	0.231	-0.117	0.519	0.031	0.017	0.031
	2.990	2.021	0.282	-0.134	0.627	0.077	0.071	0.077

References

[1] Lemmon, E. W.; Bell, I. H.; Huber, M. L.; McLinden, M. O. NIST Standard Reference Database 23: Reference Fluid Thermodynamic and Transport Properties - REFPROP. 2018; https://www.nist.gov/srd/refprop.

[2] Nguyen, H. G. T. et al. A reference high-pressure CO_2 adsorption isotherm for ammonium ZSM-5 zeolite: results of an interlaboratory study. *Adsorption* **2018**, *24*, 531–539.

[3] Nguyen, H. G. T. et al. A reference high-pressure CH_4 adsorption isotherm for zeolite Y: results of an interlaboratory study. *Adsorption* **2020**, *26*, 1253–1266.

[4] Langmuir, I. The Adsorption of Gases on Plane Surfaces of Glass, Mica and Platinum. *J. Am. Chem. Soc.* **1918**, *40*, 1361–1403.

[5] Czepirski, L.; Jagiello, J. Virial-type thermal equation of gas-solid adsorption. *Chem. Eng. Sci.* **1989**, *44*, 797–801.

[6] Nuhnen, A.; Janiak, C. A practical guide to calculate the isosteric heat/enthalpy of adsorption via adsorption isotherms in metal–organic frameworks, MOFs. *Dalton Trans.* **2020**, *49*, 10295–10307.
